# Coupled N and P cycling as driven by microbial taxa and interactions

**DOI:** 10.3389/fmicb.2025.1743883

**Published:** 2026-01-08

**Authors:** Xinyu Jiao, Yanan Wei, Yang Chen, Chaoyu Zhang, Hongmei Du, Wenjuan Yu, Hongzhang Kang

**Affiliations:** 1 Department of Landscape Architecture, School of Design, Shanghai Jiao Tong University, Shanghai, China; 2 College of Forestry and Biotechnology, Zhejiang Agriculture & Forestry University, Hangzhou, China; 3 Tianmushan Forest Ecosystem National Orientation Observation and Research Station of Zhejiang Province, Hangzhou, China; 4 Qingyuan Forest CERN, National Observation and Research Station, Shenyang, China

**Keywords:** microbial interactions, microbial taxa, microbial networks, nitrogen-phosphorus coupling, subtropical forest

## Abstract

The coupled cycling of nitrogen (N) and phosphorus (P) is fundamental to ecosystem functioning, yet the specific microbial taxa and their interactions underlying N-P coupling and decoupling remain poorly understood. Based on a natural laboratory in Yunnan with both coupled and decoupled N-P cycling, we explored bacterial, fungal, and *phoD*-harboring communities using amplicon sequencing and their relationships with N and P cycling variables. We uncovered 14 phyla and 68 genera both correlated with N and P cycling variables, identified as coupled taxa. Among them, 5 coupled phyla (*Nitrospirota*, *WPS-2*, *Mortierellomycota*, *Fungi_phy_Incertae_sedis*, and *Rozellomycota*) and 24 coupled genera (*Candidatus Koribacter*, *Candidatus Solibacter*, *A21b*, etc.) were also enriched in sites where N and P dynamics change synchronously (coupled sites), indicating a key role of these coupled taxa in promoting N-P coupling. The 11 phyla and 48 genera correlated with either N- or P-cycling variables were grouped as decoupled taxa. Moreover, the networks composed of coupled taxa (coupled networks) displayed a greater ratio of positive to negative interactions than those composed of decoupled taxa (decoupled networks). Literature confirms that potential keystone genera (*WPS-2*, *Acidibacter*, *TK10*, etc.) from the coupled network positively interacted with each other to facilitate N-P coupling while potential keystone genera (an *unclassified Subgroup_17* genus, etc.) from the decoupled network negatively interact with members to enhance N-P decoupling. These findings suggest that coupled taxa, individually and by synergistically interacting, could enhance N-P coupling whereas decoupled taxa, individually and by antagonistically interacting, might facilitate N-P decoupling. Overall, by uncovering key microbial taxa and interactions underpinning N-P coupling, our study provides a foundation for managing nutrient cycling in forest ecosystems under environmental change.

## 1 Introduction

Nitrogen (N) and phosphorus (P) are essential macronutrients critical in regulating plant growth and overall ecosystem health (Shen et al., 2019; Yu et al., 2025). The coupled cycling of N and P is crucial for ecological processes across multiple scales, from molecular to biome-wide, serving as both essential nutrients and regulators of soil fertility and microbial activity (Liang et al., 2022). However, previous research in forest ecosystems has demonstrated that N and P often exhibit asynchronous dynamics during soil development, with typically high levels of available cations but low levels of available N and P at young sites, low levels of cations and relatively high levels of N and P at intermediate-aged sites, and high levels of N but low levels of cations and P at the oldest sites (Chadwick et al., 1999). The decoupling of N and P cycles may have intensified over the past five decades due to accelerating climate change and anthropogenic disturbances (Delgado-Baquerizo et al., 2013; Peñuelas et al., 2020). The rapid rise of anthropogenic N inputs relative to P has increased global N: P ratios, while P mining and fertilization caused localized P accumulation, both exacerbating N-P imbalances (Peñuelas and Sardans, 2022; Bennett et al., 2001). Such asynchronous N-P decoupling may disrupt ecosystems by creating imbalances in nutrient ratios and negatively impact plant growth, microbial metabolism, and animal life histories (Peñuelas et al., 2013), ultimately cascading to trophic structures and ecosystem services (Yuan and Chen, 2015). Therefore, as a key player in nutrient cycling, it is vital to understand how soil microorganisms facilitate coupled N-P cycling to develop sustainable solutions, yet the specific microbial taxa involved and their complex interactions are poorly understood.

Microorganisms mediate key N (N fixation, mineralization, denitrification, etc.) and P (P transformation and mobilization) cycling processes (Hayatsu et al., 2008; Zhou et al., 2025). For example, the phyla *Nitrospirota* and *Acidobacteria* play central roles in aerobic nitrification and in P solubilization and immobilization, respectively (Li Y. et al., 2022; Mosley et al., 2024). Some microbial taxa could regulate N and P cycles simultaneously. The phylum *Proteobacteria* drive ammonia oxidation and harbor *phoD*/*phoA* genes encoding alkaline phosphatase (Dai et al., 2020; Fang et al., 2020), and the genus *Aspergillus* produce ammonium and solubilize phosphate (Ma et al., 2024; Akplo et al., 2025). Moreover, increased N availability could increase P availability via changing microbial structure and stimulating microbial growth and activity, and vice versa. For example, N addition stimulated phosphatase enzyme activity and increased the abundance of genes involved in P solubilization while P addition increased *nifH* gene abundance and biological nitrogen fixation rates (Li et al., 2020; Liu et al., 2025; Zhou et al., 2025). While it is well established that soil microbes significantly influence N and P cycles, we still know little about specific microbial taxa that facilitate the coupling of these two nutrient cycles.

Further, microbial taxa do not exist in isolation, but rather form complex networks through facilitative, competitive or neutral interactions (Ma et al., 2020). These microbial interactions greatly influence a variety of ecosystem processes associated with nutrient cycling (Jiao et al., 2021). For example, synergistic interactions of nitrifiers promoted transformation of ammonium to nitrate, whereas negative interactions among microbes involved in N-cycling limited N denitrification and anammox (Wang Z. et al., 2024). Latest studies showed that coordinated changes of functional taxa mediated coupling or decoupling of N and P cycling. Collaborations between genera *Fluviibacter* and *Sediminibacter*, both able to accumulate polyphosphate, could fuel nitrite reduction by generating ATP through anaerobic polyphosphate hydrolysis under carbon-limited conditions (An et al., 2025). The negative relationship between *Sphingomonas* participating in denitrification and *Lactobacillus* involved in P solubilization may lead to one nutrient’s cycle being favored over the other, thus disrupting the balance of N and P cycling (Zhang et al., 2021; Park et al., 2022; Liu X. Y. et al., 2023). We therefore hypothesize that N-P coupling is enhanced either by the individual functional roles of microbial taxa or by their cooperation interactions. Despite these reports, how microbial interactions influence P cycling and regulate coupled or decoupled N-P cycling remains largely overlooked.

To test our hypotheses and address these knowledge gaps, we collected soil samples from 35 sites with varying conditions of N and P cycling in central Yunnan Province, China. Importantly, this region harbors both sites where N and P change synchronously and sites where they change asynchronously, thereby providing an ideal natural laboratory to investigate the roles of microbial taxa and their interactions in N-P coupling and decoupling. Here, by linking bacterial, fungal, and *phoD*-harboring communities with seven N and P cycling variables, we aim to (i) identify key microbial taxa involved in coupled and decoupled N and P cycling, (ii) study the associations of microbial interactions with N-P coupling and decoupling. This work will enhance mechanistic understanding of ecosystem nutrient dynamics and may inform strategies for managing nutrient cycling in forested ecosystems under environmental change.

## 2 Materials and methods

### 2.1 Study site and field sampling

**Figure 1:**
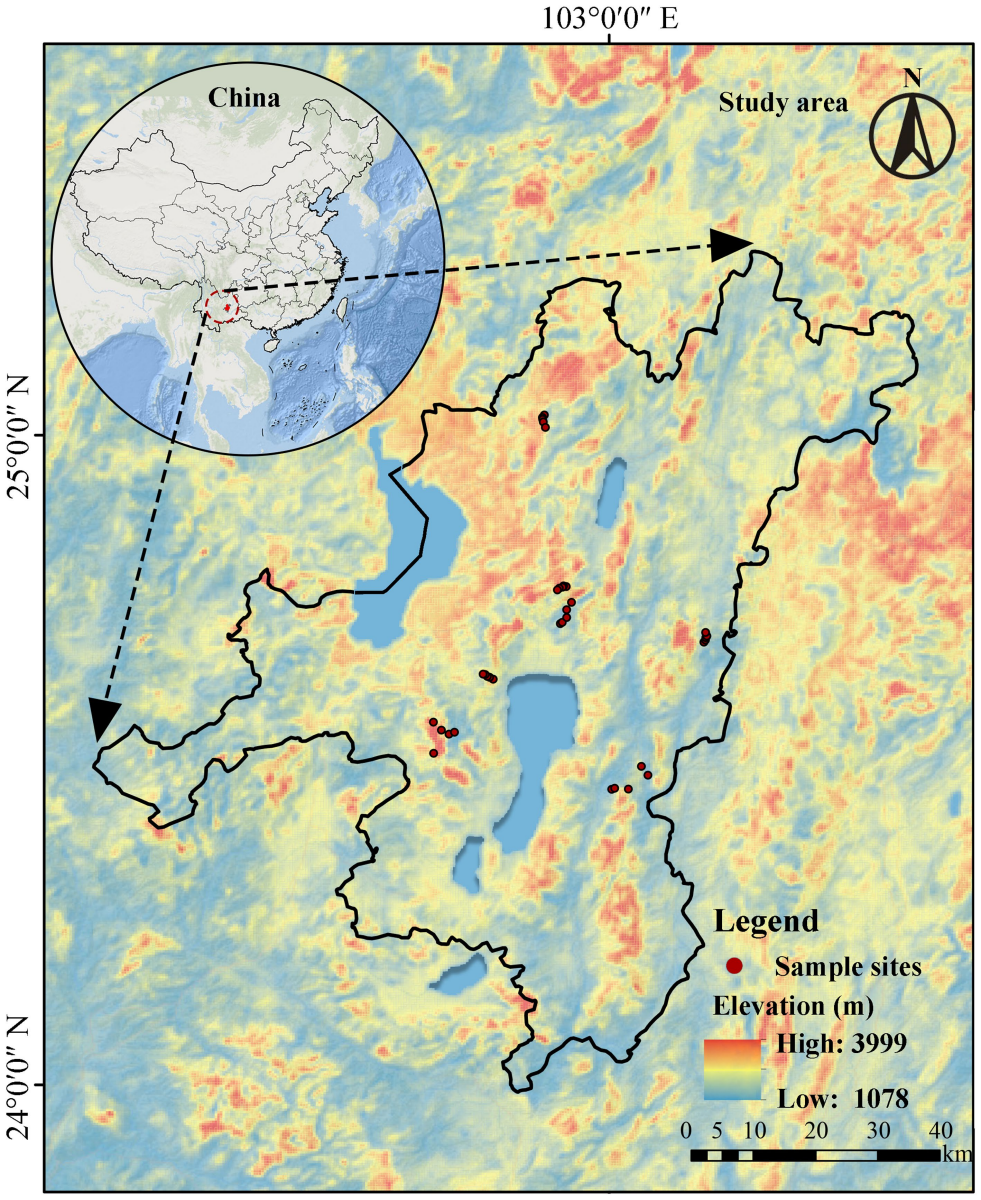
Geographical distribution of the 35 sampling sites in Yunnan.

In August 2024, 35 sites with varying P conditions were selected in central Yunnan Province, China (102°73′–103°15′E, 24°45′–25°03′N, Figure 1). The region has a subtropical monsoon climate, with a mean annual temperature (MAT) of 13.9 °C and a mean annual precipitation (MAP) of 954.7 mm, most of which falls from May through October (Zhang et al., 2019). Most soils in this region are ultisols in the U. S. Department of Agriculture classification. These sites are dominated by *Pinus yunnanensis.* At each site, after removing litter layer, nine cores were collected using a 5-cm corer at 0–10 cm depth on a S-shape transect within a 30 × 30 m^2^ area and composited to create one soil sample per site. Stem diameter at breast height at 1.3 m (DBH) was measured for representative trees within the area. Soil samples were placed in polyethylene bags, stored on ice and transferred to the laboratory within 24 h. Soils were sieved (2 mm) with any remaining visible plant material and stone removed by hand. Then, soils were subsampled and separately processed for measurements of physicochemical properties, nutrient cycling, and microbial community composition.

### 2.2 Measurements of soil physicochemical properties and nutrient cycling variables

Soil moisture was determined by comparing the weights of fresh soils before and after oven-drying at 105 °C until constant weight. The air-dried subsamples were used for the following analyses. Soil pH was measured at a soil-to-water ratio of 1:2.5 (m/v). Soil particles were classified into silt, clay, and sand using Bouyoucos hydrometer method (Bouyoucos, 1962). Soil organic carbon (SOC) and total N (TN) contents were determined using a Vario EL III elemental analyzer (Elementar Analysensysteme GmbH, Germany). Total P (TP) were extracted by perchloric and nitric acids and analyzed by an iCAP6300 inductively coupled plasma spectrometer (ICP, Thermo Fisher, America). Available P (AP), reflecting organic P that could easily be mineralized from soil organic matter and inorganic P that could be easily released from minerals to be available for plants and microbes (Zhu et al., 2018), was also analyzed on the ICP following extraction by 0.05 M hydrochloric acid and 0.025 M sulfuric acid using the Mehlich 1 method (Mehlich, 1953). To measure activity of N-acetyl-*β*-glucosaminidase (NAG), a key enzyme in N mineralization from soil organic matter (Sinsabaugh et al., 2008), 1 g of soil was incubated at 37 °C for 60 min and the released 4-nitrophenol from added substrate (4-Nitrophenyl N-acetyl-β-D-glucosaminide) was photometrically quantified at absorbance 400 nm following formation of the yellow derivative (Eivazi and Tabatabai, 1988). To measure activity of alkaline phosphatase (AKP), a proxy to evaluate the mineralization of organic P to bioavailable inorganic P in both acidic and alkaline soils (Bergkemper et al., 2016), 1 g of soil was incubated at 37 °C for 24 h and the released phenol from added substrate (disodium phenyl phosphate) was photometrically quantified at absorbance 570 nm following formation of the red derivative quinone (Kang et al., 2018). Acidic phosphatase activity was also measured but not presented, as it generally had weak relationships with microbial taxa.

Besides the above-measured TN, TP, AP, NAG, and AKP, we added two additional variables to indicate soil nutrient cycling: inorganic N (N_inorg_) and net nitrogen mineralization after 28 days (net N_min_). We extracted ammonium and nitrate from field-moist subsamples using 1 M potassium chloride. The ammonium N concentration was analyzed photometrically at 630 nm. Nitrate was reduced to nitrite using a cadmium reduction method, and the resulting nitrite N concentration was quantified photometrically at 543 nm following diazo salt formation (Ministry of Environmental Protection of the People's Republic of China, 2012). Net N mineralization was determined by calculating the difference in ammonium-N plus nitrate-N pools (inorganic N) between the initial samples and those after 28 days of incubation.

### 2.3 Amplicon sequencing and bioinformatics of 16S rRNA, ITS rRNA, and *phoD* genes

To investigate community composition of bacteria, fungi, and *phoD*-harboring microorganisms, soil DNAs were extracted using OMEGA Soil DNA Kit (D5635-02). The quantity and quality of extracted DNAs were measured using a NanoDrop NC2000 spectrophotometer (Thermo Fisher, USA) and agarose gel electrophoresis, respectively. The V3-V4 region of bacterial 16S rRNA gene was amplified using the 338 F (5’-ACTCCTACGGGAGGCAGCA-3′)/806 R (5’-GGACTACHVGGGTWTCTAAT-3′) primer (Mori et al., 2014). The ITS1 region of fungal ITS rRNA gene was amplified using the ITS5F (5’-GGAAGTAAAAGTCGTAACAAGG-3′)/ITS2R (5’-GCTGCGTTCTTCATCGATGC-3′) primer (Li P. et al., 2022). The *phoD* gene was amplified using the ALPS-F730 F (5’-CAGTGGGACGACCACGAGGT-3′)/ALPS-1101 R (5’-GAGGCCGATCGGCATGTCG-3′) primer (Luo et al., 2017). Following purification and quantification of PCR amplicons, amplicons were pooled in equal amounts. Sequencing (2 × 250 bp) of pooled bacterial, fungal, and *phoD* amplicons was performed on an Illumina MiSeq platform with Miseq Reagent Kit v3 at Shanghai Personal Biotechnology Co., Ltd. (Shanghai, China). All raw sequences were deposited in the NCBI Sequence Read Archive under accession number PRJNA1328234 (16S), PRJNA1328344 (ITS), and PRJNA1330695 (*phoD* gene).

The obtained sequences were analyzed using the following steps. First, low-quality sequences and chimeras of 16S and ITS rRNA genes were removed using DADA2 (Callahan et al., 2016), followed by clustering of high-quality sequences into amplicon sequence variants (ASVs); low-quality sequences and chimeras of *phoD* gene were removed using Vsearch, and the remaining sequences were clustered into operational taxonomic units (OTUs) based on 97% sequence similarity, expressed also as ASVs below for convenience. Second, representative sequences of bacterial and fungal ASVs were used for taxonomic assignment from kingdom to species based on the Silva (version 138) and UNITE (version 9) databases, respectively; taxonomic classification of *phoD*-harboring ASVs from kingdom to species was conducted using the NCBI Nucleotide database. Third, ASVs comprising < 0.001% of total sequences across all samples were removed. A rarefied ASV table was obtained by averaging 100 evenly resampled ASV subsets under the 90% of the minimum sequencing depth. Bacterial and *phoD*-harboring ASVs present in at least 10% of the samples and fungal ASVs that had at least 10 sequences across all samples were retained. For bacteria, there were 959–2,359 ASVs (mean = 1864) and 35,454–64,657 sequences (mean = 55,877) per sample; for fungi, there were 344–527 ASVs (mean = 428) and 101,457–101,858 sequences (mean = 101,666) per sample; for *phoD*-harboring bacteria, there were 592–2,445 ASVs (mean = 1,577) and 27,902–40,310 sequences (mean = 36,816) per sample.

### 2.4 Statistical analyses

We performed all statistical analyses in R software version 4.4.2 (R Core Team, 2024). Overall community composition of bacteria, fungi, and *phoD*-harboring bacteria were visualized using principal coordinate analysis (PCoA) based on Bray-Curtis distances using R package “vegan.” Then we used the “envfit” function to explore relationships among microbial community composition and N- and P-cycling variables.

**Figure 2:**
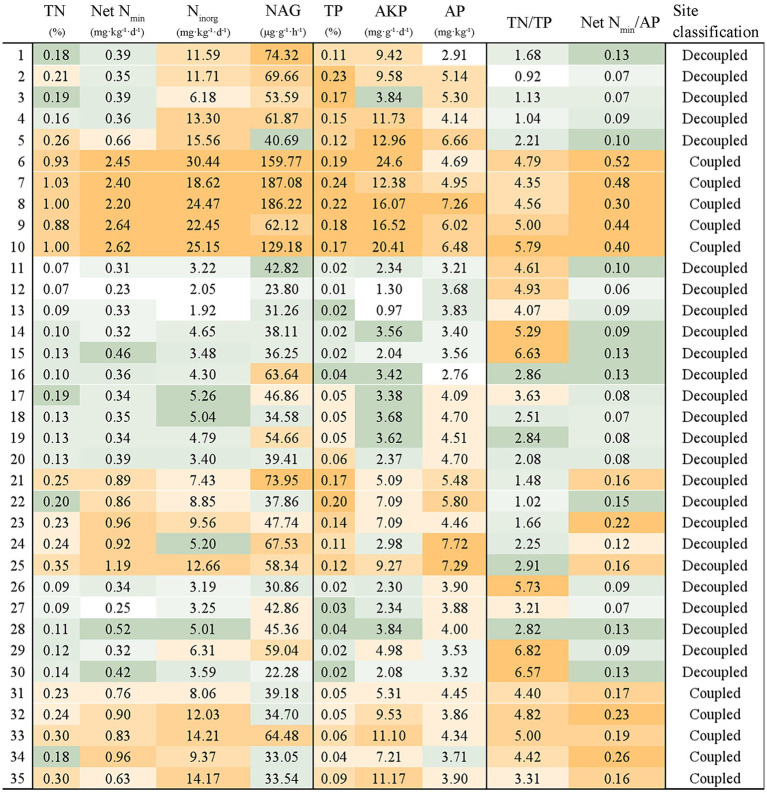
The investigated N and P cycling variables at 35 *Pinus yunnanensis* sites in Yunnan. Orange and green grids represent relatively high and low concentrations. Sites 6–10 and 31–35 with TN/TP > 3 and net N_min_/AP > 0.16 were identified as N-P coupled sites while the other sites were identified as N-P decoupled sites due to either low TN/TP (< 3) or net N_min_/AP (< 0.16) or both.

Notably, soil TN/TP and net N_min_/AP are commonly used to reflect coupling degree of soil N and P cycling (Cheng et al., 2020; Liu et al., 2024; Huang et al., 2025). Since there were no sites with fast N cycling and slow P cycling in this study, we defined sites (6–10 and 31–35) with relatively high TN, TP, net N_min_, and AP, together with relatively high TN/TP (> 3) and net N_min_/AP (> 0.16) as “coupled sites” (Figure 2). Since there are no single universal threshold exists for coupled N-P cycling and thresholds vary across ecosystems (Zhang et al., 2013), the cycling of N and P in these sites were considered relatively fast and well-synchronized due to these relatively high values. The remaining sites (1–5 and 11–30) with either low TN/TP (< 3) or net N_min_/AP (<0.16) or both were classified as “decoupled” as they fell into the following two scenarios: ① N cycling was slow yet P cycling was fast, or ② the cycling of N and P were both slow. This classification accounts for sites with relatively asynchronous cycling of N and P as well as sites where cycling rates were too low to be considered effectively coupled.

To examine microbial taxa important for coupled vs. decoupled N and P cycling, correlations of abundances of major phyla and genera (relative abundance > 0.1%) with N- and P-cycling variables were performed, followed by Bonferroni adjustment for multiple comparisons. Microbial taxa that had relationships with either N- or P-cycling variables were grouped as those important for decoupled N and P cycling (decoupled taxa). Microbial taxa that had consistently positive or negative relationships with both N- and P- cycling variables were grouped as those important for coupled N and P cycling (coupled taxa). A regression model was further used to explored significant (*p* < 0.05) relationships of the coupled taxa with net N_min_/AP, followed by Bonferroni adjustment, since net N_min_/AP could more directly reflect coupled process of soil N and P cycling than TN/TP.

To further explore interactions among the coupled and decoupled phyla, respectively, we selected major phyla (relative abundance > 0.1%) occurring in over half of the 35 samples to construct undirected microbial co-occurrence networks, using the ‘igraph’ package. Similar steps were performed to explore interactions among the coupled and decoupled genera, respectively. Following multiple test corrections using the FDR-BH method, only robust (Pearson |r| > 0.6) and significant (*p* < 0.05) correlations were incorporated into the network analyses (Long et al., 2025). A total of 14 (7 bacterial and 7 fungal) phyla and 64 (34 bacterial, 25 fungal and 5 *phoD*-harboring bacterial) genera were included in the networks constructed using the coupled taxa (coupled networks), respectively. A total of 11 (6 bacterial, 1 fungal and 4 *phoD*-harboring bacterial) phyla and 44 (29 bacterial, 9 fungal and 6 *phoD*-harboring bacterial) genera were included in the networks constructed using the decoupled taxa (decoupled networks), respectively. Following exploration and visualization of the networks in Gephi (version 0.9.2), we analyzed the following network topological properties: node, edges, potential keystone taxa, connectance, ratio of positive to negative edges. After standardization, a combined score of high degree centrality, high closeness centrality, and low betweenness centrality was used for determining a putative keystone taxon with a threshold ≥ 1 (Banerjee et al., 2018; Sun et al., 2024). The connectance, determined by the ratio of actual to total possible edges among nodes, is a way to quantify how densely connected a network is (Karimi et al., 2017).

## 3 Results

### 3.1 Soil physicochemical properties and nutrient cycling variables

Soil pH (4.73–7.87) and TP (0.01–0.24%) displayed a broad range across sampling sites, with TP concentration ranging from extremely P-lacking to extremely P-rich conditions according to the soil nutrient classification standards from the second national soil survey (National Soil Survey Office, 1990) (Supplementary Table S1; Figure 2). Soil moisture content (58.62–69.14%), SOC (13.05–15.62%) and TN (0.07–1.03%) concentrations at sites 6–10 were much higher than the other sites. Further, the net N_min_/AP and TN/TP were both high at sites 6–10 and 31–35, suggesting coupled N and P cycling at these sites.

### 3.2 Relationships of overall microbial community composition with nutrient cycling variable

**Figure 3:**
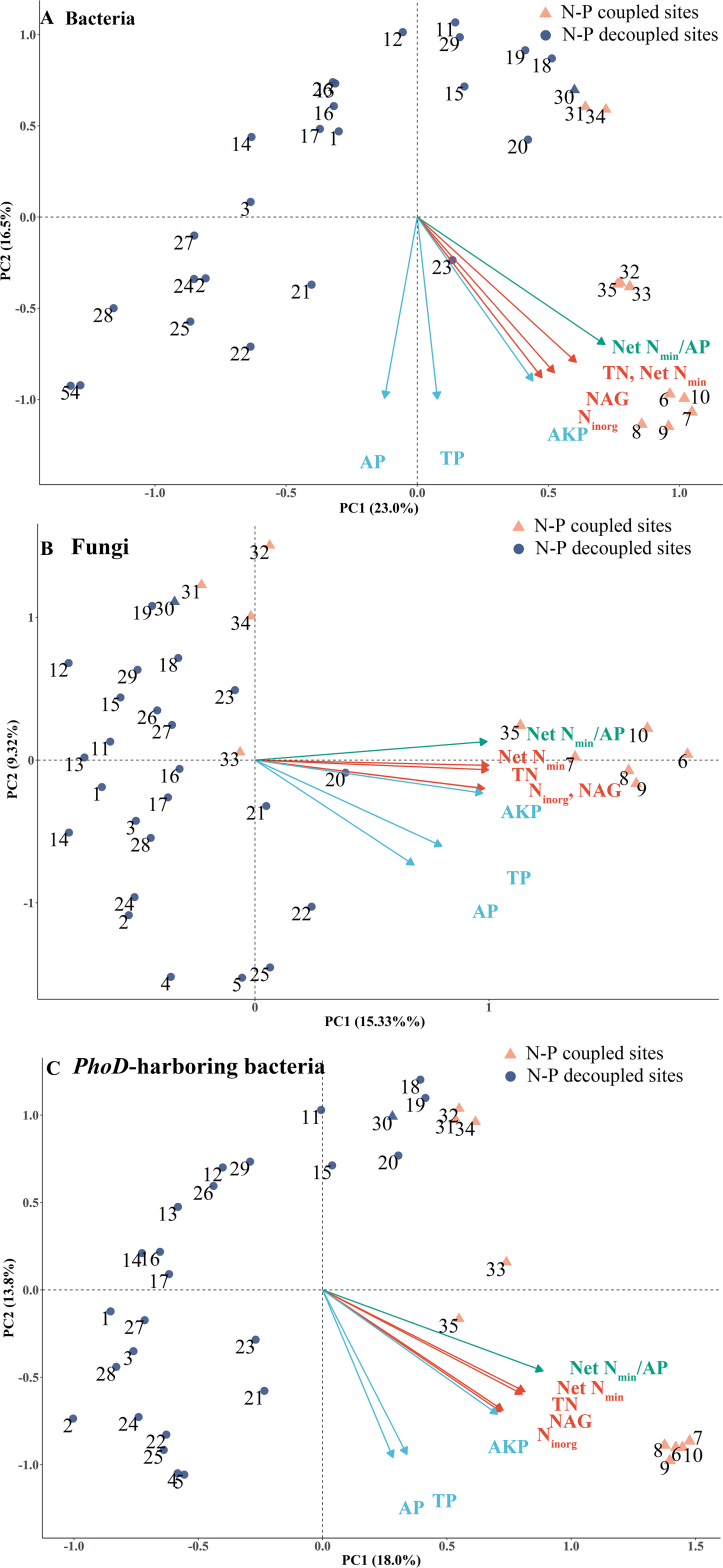
Principal coordinate analysis (PCoA) demonstrating overall differences in **(A)** bacterial, **(B)** fungal, and **(C)** *phoD*-harboring bacterial community composition among sites. Soil N-cycling variables (TN, NAG, net N mineralization, and inorganic N) shown in red arrows, soil P-cycling variables (TP, AP, and AKP) shown in blue arrows, and the net N_min_/AP shown in green arrows were all significantly (*p* < 0.05) related to the first two axes. Orange triangles represent N-P coupled sites, whereas blue circles represent N-P decoupled sites.

The PCoA showed that the community composition of bacteria, fungi, and *phoD*-harboring bacteria from sites 6–10 differed greatly from other sites (Figures 3A–C). The first two axes explained 39.5, 26.4, and 31.8% of the variations in bacterial, fungal, and *phoD*-harboring bacterial community composition, respectively, and had significant relationships with all the seven nutrient cycling variables (*p* < 0.05). N-cycling variables clustered more closely than P-cycling ones. All N- and P-cycling variables and net N_min_/AP pointed toward sites 6–10 and sometimes sites 31–35, suggesting of specific microbial taxa responsible for faster and more coupled N and P cycling at these sites.

Ten bacterial phyla (*Acidobacteriota* 31.7%, *Proteobacteria* 30.9%, *Chloroflexi* 10.8%, *Actinobacteriota* 8.9%, *Verrucomicrobiota* 3.8%, *Gemmatimonadota* 3.1%, *Bacteroidota* 2.7%, *Myxococcota* 1.7%, *Methylomirabilota* 1.7% and *RCP2*-*54* 1.0%), four fungal phyla (*Ascomycota* 51.5%, *Basidiomycota* 43.6%, *Mortierellomycota* 3.2% and *Mucoromycota* 0.6%) and two *phoD*-harboring bacterial phyla (*Pseudomonadota* 95.7%*, Actinomycetota* 3.0%) accounted for 96.3, 98.9 and 98.7% of total sequences across all soil samples, respectively (Supplementary Figure S1). Relative abundances of bacterial and fungal phyla varied among sites. For bacteria, *Nitrospirota* (0.5% averaged) and *WPS*-*2* (2.0%) were generally more abundant at the coupled sites 6–10 and 31–35 compared to the other sites (Supplementary Figure S1a). For fungi, *Mortierellomycota* (5.8%) and *Rozellomycota* (0.1%) were more abundant at the coupled sites (Supplementary Figure S1b).

### 3.3 Relationships between N-P cycling and microbial taxa

**Figure 4:**
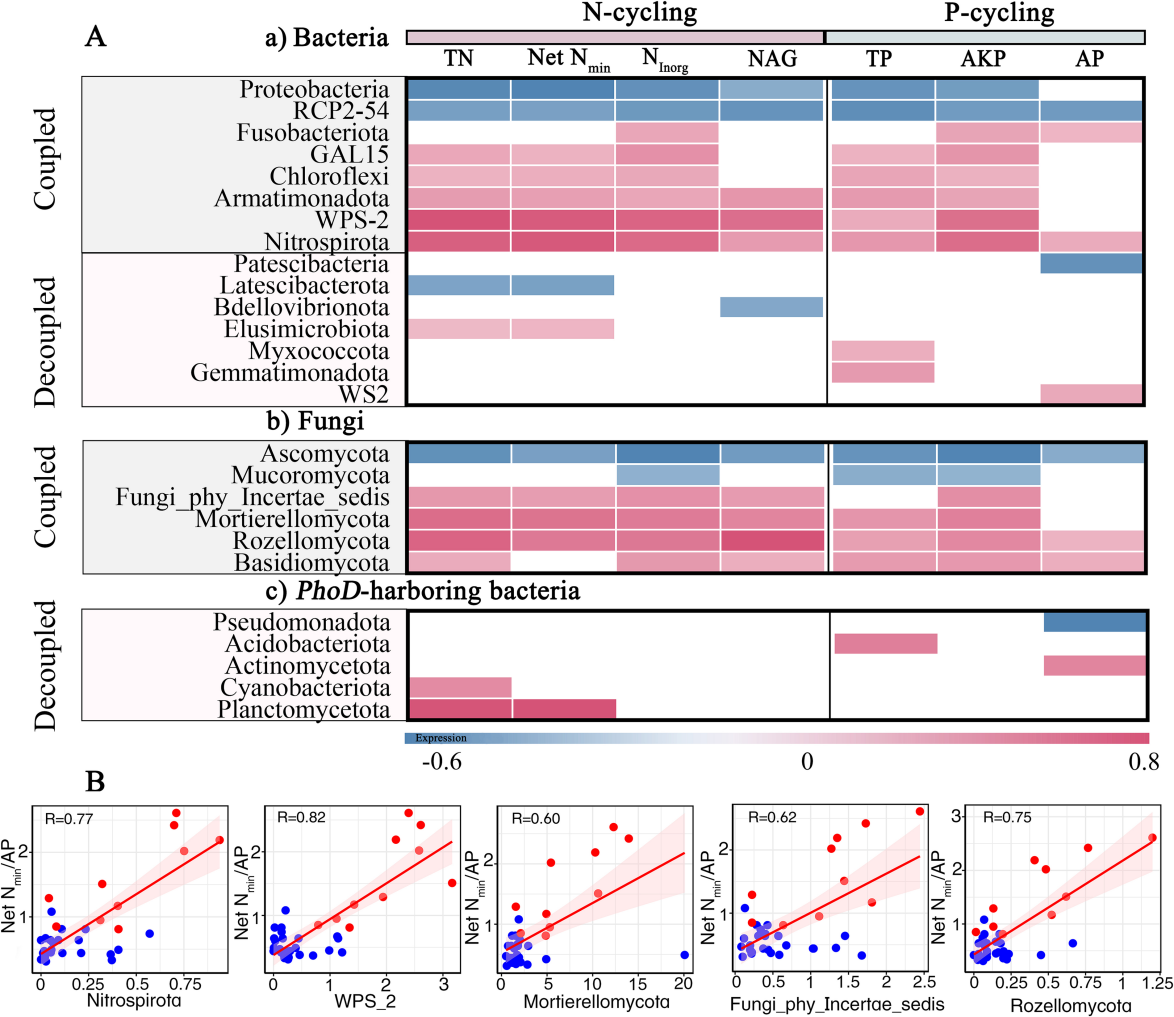
**(A)** Pearson correlations of N- and P-cycling variables with major **(a)** bacterial, **(b)** fungal, and **(c)** *phoD*-harboring bacteria phyla. Significant (*p* < 0.05) correlations are shown in grids. Coupled means coupled N and P cycling as driven by microbial taxa, as indicated by consistently positive (pink) or negative (blue) relationships of microbial taxa with N and P cycling variables. Decoupled means decoupled N and P cycling as driven by microbial taxa, as indicated by relationships of microbial taxa with either N or P cycling variables. **(B)** Significant (*p* < 0.05) regressions between net N_min_/AP ratio and relative abundances (%) of N-P coupled phyla identified in **(A)**. Shaded sections are the 95% confidence intervals of the regression models. Red and blue dots represent coupled and decoupled sites, respectively.

**Figure 5:**
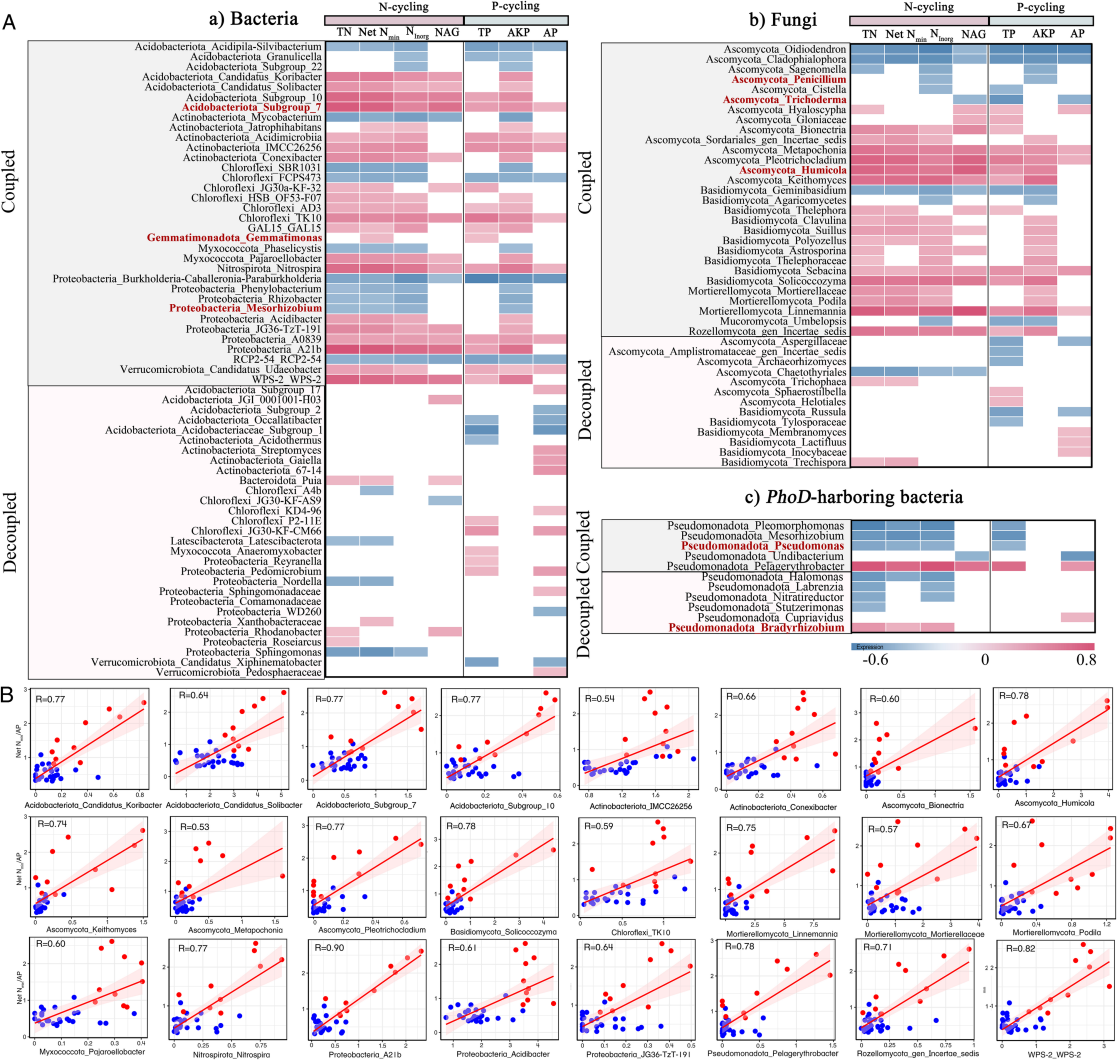
**(A)** Pearson correlations of N- and P-cycling variables with major **(a)** bacterial, **(b)** fungal, and **(c)** *phoD*-harboring bacteria genera. Significant (*p* < 0.05) correlations are shown in grids. Coupled means coupled N and P cycling as driven by microbial taxa, as indicated by consistently positive (pink) or negative (blue) relationships of microbial taxa with N and P cycling variables. Decoupled means decoupled N and P cycling as driven by microbial taxa, as indicated by relationships of microbial taxa with either N or P cycling variables. Names of phyla and lowest taxonomic levels that a microbial genus could be classified to are presented. Red highlights phosphorus-solubilizing microorganisms. **(B)** Significant (*p* < 0.05) regressions between net N_min_/AP ratio and relative abundances (%) of N-P coupled genera identified in **(A)**. Shaded sections are the 95% confidence intervals of the regression models. Red and blue dots represent coupled and decoupled sites, respectively.

**Figure 6:**
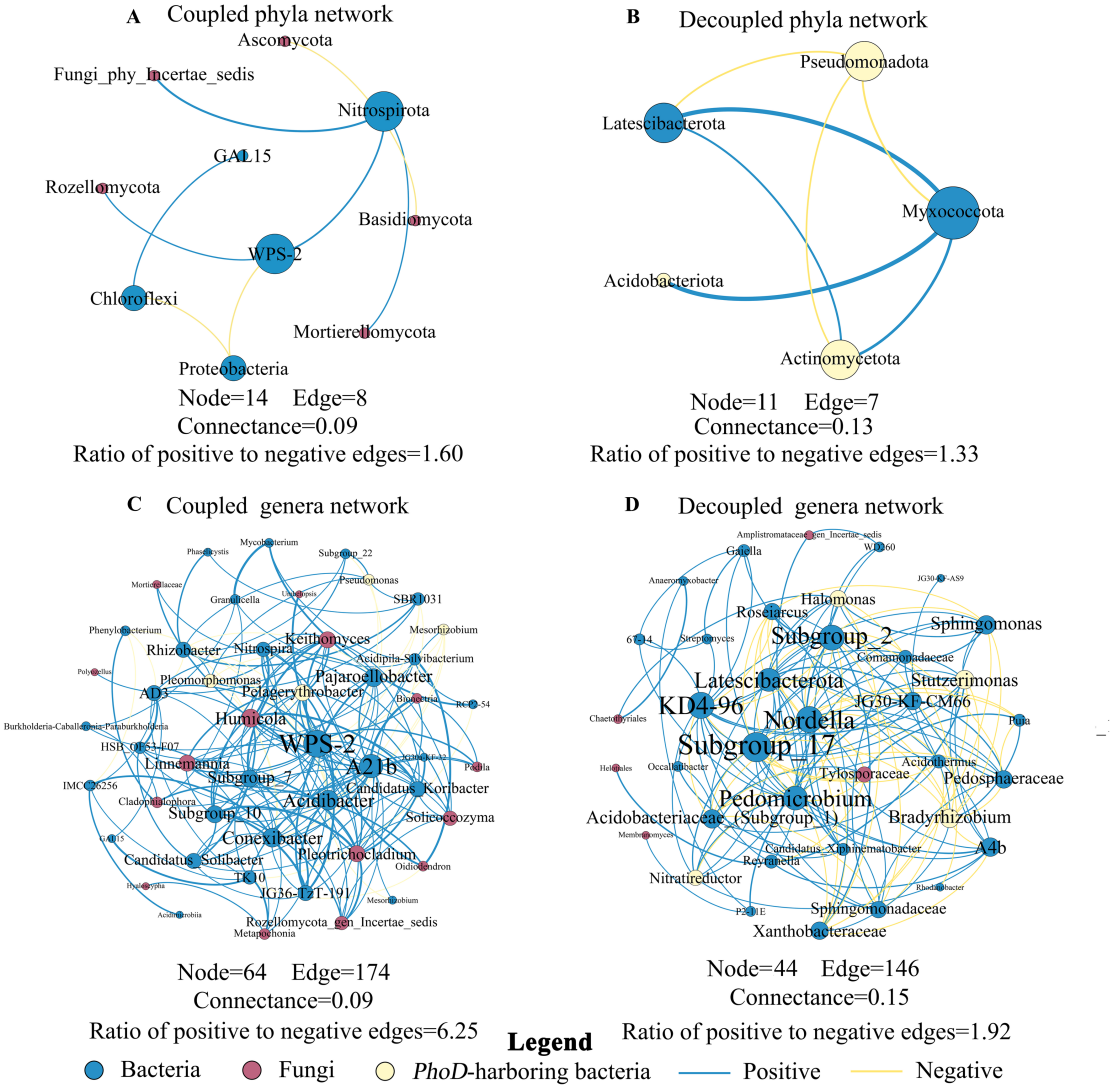
Microbial co-occurrence networks among the high-frequency **(A)** coupled phyla, **(B)** decoupled phyla, **(C)** coupled genera, and **(D)** decoupled genera. Blue, red, and yellow dots represent bacterial, fungal, and *phoD*-harboring bacterial nodes, respectively. Blue and yellow lines represent positive and negative correlations, respectively. The larger nodes were often defined as keystone taxa.

Microbial phyla and genera exhibited significant (*p*<0.05) correlations with N and/or P cycling variables (Figures 4–6), indicating their roles in driving coupled and decoupled N and P cycling. Seven bacterial and five *phoD*-harboring phyla only had relationships with either N- or P-cycling variables (Figure 4A). These phyla might promote decoupled N and P cycling and were abbreviated as “decoupled phyla.” In contrast, bacterial phyla *Proteobacteria* and *RCP2*-*54* showed negative correlations with both N and P cycling variables while *Fusobacteriota*, *GAL15*, *Chloroflexi*, *Armatimonadota*, *WPS*-*2*, and *Nitrospirota* showed positive correlations; fungal phyla *Basidiomycota*, *Rozellomycota, Mortierellomycota*, and *Fungi_phy_Incertae_sedis* were positively correlated with N and P cycling variables while *Ascomycota* and *Mucoromycota* were negatively correlated. These phyla might drive coupled N and P cycling and were sometimes abbreviated as “coupled phyla.” Further, five of these coupled phyla (*Nitrospirota*, *WPS-2*, *Mortierellomycota*, *Fungi_phy_Incertae_sedis*, *and Rozellomycota*) were positively related to net N_min_/AP and generally more abundant in the coupled sites than in the decoupled sites (Figure 4B), suggesting that enrichment of the coupled phyla was a reason for coupled N and P cycling in sites 6–10 and 31–35.

At the genus level, 29 bacterial, 13 fungal, and 6 *phoD*-harboring genera only had significant relationships with either N- or P-cycling variables (Figure 5). These genera might promote decoupled N and P cycling and were abbreviated as “decoupled genera.” The 13 fungal genera were predominantly associated with P-cycling variables while the 6 *phoD*-harboring genera mainly with N-cycling variables. In contrast, 34 bacterial (22 positive and 12 negative), 29 fungal (20 positive and 9 negative), and 5 *phoD*-harboring (1 positive and 4 negative) genera were found to exhibit correlations with both N and P cycling variables. These genera might drive coupled N and P cycling and were abbreviated as “coupled genera.” Further, 24 of these coupled genera were positively related to net N_min_/AP and generally more abundant in the coupled sites compared with decoupled sites (Figure 5B), suggesting that these genera could play a role in promoting N-P coupling at the coupled sites.

Notably, microbial phyla and genera within the phyla sometimes showed inconsistent relationships with N- and P-cycling variables. For example, *Ascomycota* was negatively correlated with both N- and P-cycling variables, yet an unclassified *Aspergillaceae* genus from the phylum was only negatively correlated with P-cycling variables (Figures 4, 5). *Basidiomycota* was positively correlated with N- and P-cycling variables, yet *Trechispora* within the phylum only displayed positive relationships with N-cycling variables. Phosphate-solubilizing microorganisms were not always related to P cycling: *Penicillium*, *Pseudomonas, Trichoderma* and *Mesorhizobium* were negatively correlated with both N- and P-cycling variables; whereas *Bradyrhizobium* only showed positive correlations with N cycling variables. Roles of these genera in N and P cycling were discussed below.

### 3.4 Networks constructed using coupled vs. decoupled taxa

We further constructed co-occurrence networks for abundant phyla and genera that were possibly involved in coupled N and P cycling (Figures 4, 5) and occurred in over half of the 35 samples, abbreviated as “coupled phyla network” and “coupled genera network,” respectively (Figures 6A,C). Similarly, we constructed co-occurrence networks for abundant phyla and genera that were possibly involved in decoupled N and P cycling (Figures 4,5) and occurred in over half of the samples, abbreviated as “decoupled phyla network” and “decoupled genera network” (Figures 6B,D). Co-occurrence patterns and topological indices differed between coupled and decoupled networks, at both phylum and genus levels. The coupled networks exhibited a higher complexity (14 nodes and 8 edges at the phylum level; 64 nodes and 174 edges at the genus level) than the decoupled ones (11 nodes and 7 edges at the phylum level; 44 nodes and 146 edges at the genus level). However, the decoupled networks (0.13 at the phylum level; 0.15 at the genus level) had higher connectance than the coupled ones (0.09 at the phylum level; 0.09 at the genus level), indicating more active interactions among decoupled taxa.

The networks were dominated by bacterial taxa, with the edges of bacteria-bacteria (B-B), bacteria-fungi (B-F) and bacteria–*phoD*-harboring bacteria (B-P) constituting the majority of the edges (90.0 and 86.7% at the genus and phylum levels, respectively, Supplementary Table S2). Edges of the coupled networks were predominantly positive, with a ratio of positive to negative edges being 1.60 and 6.25 at the phylum and genus levels, respectively. While edges of the decoupled networks were still mostly positive, the ratio of positive to negative edges decreased to 1.33 (phylum) and 1.92 (genus), respectively. This finding suggests of a decline in cooperative interactions and an increase in competitive ones among decoupled taxa, relative to coupled taxa, at both phylum and genus levels.

At the phylum level, *WPS-2* (2 positive and 1 negative edges) in the coupled network and *Myxococcota* (3 positive and 1 negative) in the decoupled network were identified as putative keystone taxa. At the genus level, *WPS-2* genus from *WPS-2* (19 positive and 4 negative), *A21b* from *Proteobacteria* (20 positive), *Acidibacter* from *Proteobacteria* (9 positive and 6 negative), *TK10* from *Chloroflexi* (6 positive and 2 negative), *AD3* from *Chloroflexi* (8 positive and 2 negative), an unclassified *Subgroup_7* genus from *Acidobacteriota* (12 positive) and *Conexibacter* from *Actinobacteriota* (10 positive and 4 negative) in the coupled network were identified as potential keystone taxa. An *unclassified Subgroup_17* genus *from Acidobacteriota* (13 positive and 8 negative), *Nordella* from *Proteobacteria* (11 positive and 7 negative), *Pedomicrobium* from *Proteobacteria* (12 positive and 4 negative), an *unclassified Subgroup 2* genus from *Acidobacteriota* (7 positive and 10 negative), *Latescibacterota* genus from *Latescibacterota* (11 positive and 4 negative), *KD4-96* genus from *Chloroflexi* (14 positive and 4 negative), and *JG30-KF-CM66* from *Chloroflexi* (7 positive and 3 negative) in the decoupled network were identified as potential keystone taxa. Similarly, keystone genera in the decoupled network generally had higher ratios of negative to positive edges than those in the coupled network, indicating of increasingly competitive and antagonistic interactions among the keystone genera and their partners in the decoupled network.

## 4 Discussion

Generally, the soils with TP < 0.2 g·kg^−1^ are extremely P-deficient, whereas soils with TP > 1 g·kg^−1^ are classified as P-rich according to the Second National Soil Survey of China (National Soil Survey Office, 1990). The sites in our study were selected to represent varying P conditions, and two threshold were further proposed in this study to interpret the observed coordination between N and P cycling under different soil conditions. Since the thresholds for the coupled cycling of N and P are ecosystem-dependent (Zhang et al., 2013), the classification (TN/TP > 3 and net N_min_/AP > 0.16 were defined as coupled sites, TN/TP < 3 or net N_min_/AP < 0.16 or both were defined as decoupled sites) adopted herein is empirically derived from our data; however, it ought to be considered system-specific rather than a universally standard. The mean TN/TP ratio of coupled sites in this study (4.7) was slightly higher than the average (4.2) for China’s topsoil (Tian et al., 2010), yet lower than mean for tropical forest (5.72) at global scale (Xu et al., 2013). In subtropical riparian wetland, TN/TP ratios generally covered the ranges from 0.43 to 1.48, and higher TN/TP ratios were associated with low soil microbial activity (Yu et al., 2020; Wang et al., 2015). In sandy ecosystem, Dong et al. (2023) reported that lower TN/TP ratio (3.8) generally fail to sustain synchronous N and P turnover rates. Some studies showed that soil enzyme activity significantly positive affected TN/TP ratio, and that increases in species richness and microbial community complexity could promote P accumulation in sandy ecosystem (Wang et al., 2025; Liu et al., 2022). These cross-system differences highlight that the thresholds identified in our study are context-dependent.

### 4.1 Coupled N and P cycling as driven by microbial taxa

The underlying microbial taxa driving coupled N and P cycling remain largely unexplored. In this study, we uncovered five coupled phyla and a few coupled genera that were positively related to N- and P cycling-variables as well as net N_min_/AP (Figures 4, 5). General enrichment of these taxa in the N-P coupled sites (6–10 and 31–35) was probably an important reason for coupled N and P cycling in these sites. Previous literature confirmed roles of these coupled phyla in facilitating both N and P cycling. *Nitrospirota* is well known for its central role in N cycling, including many *Nitrosospira* species containing *NxrA* (encoding nitrite oxidoreductase) and *amoB* (encoding ammonia monooxygenase) genes (Mosley et al., 2024). Meanwhile, *Nitrospirae* from *Nitrospirota* was a large family carrying *ppx* genes dissolving inorganic P (Liu et al., 2025). The phylum *WPS-2* facilitated decomposition of soil organic P and contained *nifH* (N fixation) homologs (Pessi et al., 2024; Cheng et al., 2025). *Mortierlla* from *Mortierellomycota* could dissolve insoluble P (Sang et al., 2022) and had positive relationships with soil heterotrophic nitrification rates and soil available P (Zhang et al., 2020; Zhu et al., 2022). *Rozellomycota* abundance was positively correlated with inorganic P and increased with N addition (Jian et al., 2025).

We also uncovered a few coupled genera enriched in coupled sites, and previous literature confirmed their roles in facilitating both N and P cycling. Consistent with our result, an unclassified *Subgroup_7* genus from *Acidobacteriota* was positively correlated with inorganic N and available P, via promoting conversion of organic P to inorganic P (Wang et al., 2022; Lv et al., 2024). *Candidatus Koribacter* and *Candidatus Solibacter* also from *Acidobacteriota* participated in not only P cycling (phosphate-specific transportation, alkaline phosphatase, and *gcd* gene encoding enzymes for inorganic P solubilization), but also N cycling (reduction of nitrate, nitrite, and possibly nitric oxide; Lacerda-Júnior et al., 2019; Yu et al., 2021). *A21b* from *Proteobacteria* could assimilate and release pyruvate to participate in P conversion and dissolve bound P in soil (McIlroy et al., 2015; Wang X. et al., 2024), and explained variation in *nirS* (nitrite reductase) and *nosZ* (nitrous oxide reductase) gene abundances (Truu et al., 2020). *Acidibacter* from *Proteobacteria* participated in P cycling by their positive correlations with alkaline phosphatase and indirectly by reducing Fe (III) to Fe (II) during which P was solubilized from iron minerals, and the relative abundance of the *Acidibacter* increased with high N addition (Nie et al., 2018; Wang H. et al., 2023; Ren et al., 2025). *Humicola* from *Ascomycota* could dissolve and convert ineffective P into available P and participated in N cycling by producing NAG enzyme and showing negative correlations with ammonium (Mahmoud and Narisawa, 2013; Chen et al., 2023; Fang et al., 2024). *Conexibacter* from *Actinobacteriota* have been reported to enhance ammonia oxidation and showed negative relationships with soil P pools (Jien et al., 2021; Wu et al., 2022). *Solicoccozyma* from *Basidiomycota* showed positive correlations with TN, responded positively to P addition and could solubilize inorganic phosphate (Stosiek et al., 2019; Cheng et al., 2022; Wang et al., 2022); Roles of *TK10* from *Chloroflexi* in N cycling and *Linnemannia* from *Mortierellomycota* in P cycling have been reported (Mehrshad et al., 2018; Wu et al., 2022). Our findings show that they might promote coupled N and P cycling. Interestingly, we found *Chloroflexi* also showed variation across sites, likely influenced by differences in soil pH and moisture among environment, meanwhile high P also increased its abundance, this finding is in line with previous findings (Yun et al., 2016; Zhang C. et al., 2024).

Moreover, seven of the coupled genera have been identified as phosphate-solubilizing microorganisms (PSMs; Figure 5), playing a crucial role in driving P cycling and facilitating plant P uptake (Pang et al., 2024). Three PSMs (*Gemmatimonas*, *Humicola* and an unclassified *Subgroup_7 genus* from *Acidobacteriota*) showed positive relationships not only with available P and TP but also with nitrate N, reflecting potentials of PSMs in driving N cycling and consistent with previous studies (Li et al., 2021). There are two ways for PSMs to participate in N cycling. First, PSMs can indirectly release P through inorganic acids produced during N fixation (Tao and Gao, 2023). Second, some PSMs solubilize bound P via excretion of protons produced during ammonium assimilation instead of organic acids (Sharma et al., 2013; Pang et al., 2024; Ahash et al., 2025). Notably, the other four PSMs (*Penicillium*, *Trichoderma*, *Mesorhizobium,* and *Pseudomonas*) showed significant yet negative correlations with P-cycling variables, consistent with previous studies (Lang et al., 2022). The specific reasons need further study.

Taken together, most of the statistically identified taxa had been previously reported to facilitate both N and P cycling. Enrichment of many of the taxa could facilitate coupled N-P cycling at the coupled sites. Roles of the other coupled taxa, e.g., *Keithomyces* from *Ascomycota,* in N and P cycling, need more evidence in the future. The coupled taxa might drive coupled N and P cycling or just respond to increased availability of N and P, and thus specific contributions of these taxa in promoting soil N and P cycling need further exploration.

### 4.2 Coupled N and P cycling as driven by microbial interaction

Beyond individual taxa, interactions among taxa could be another important reason to facilitate coupled N-P cycling. An increasing number of studies suggests that positive and negative connections among microbial taxa can indicate competitive and cooperative interactions, respectively (Chen et al., 2022; Zhang H. et al., 2024). Our study revealed that putative keystone taxa of coupled network (*WPS-2*, *A21b*, *Acidibacter*, *TK10*, *AD3*, an *unclassified Subgroup_7* genus, and *Conexibacter*) might facilitate N-P coupling independently (Figure 5). Moreover, their cooperative interactions could strengthen the coupling by promoting each other’s growth via feeding essential substrates and creating favorable conditions (Figures 5, 6). For example, *WPS-2* prefer peptides and amino acids as primary nutrient sources (Ji et al., 2021), which could be contributed by *TK10* during their degradation of protein (Feng et al., 2025). *WPS-2* bacteria are frequently found in acidic soils, which could be promoted by *Acidibacter* by acidifying soils (Wang F. et al., 2023). Besides growth promotion, the potential keystone taxa cross-fed essential substrates in N and P cycling. For example, *Conexibacter* utilized inorganic phosphorus to generate pyruvate, while *A21b* assimilated pyruvate to release inorganic phosphorus. In addition, *Conexibacter* reduced nitrate to nitrite, which could subsequently be used by *A21b* and *AD3* to produce nitrate (Liu X. et al., 2023; Miralles et al., 2023; Zhu et al., 2024). Thus, we conclude that cooperative interactions among potential keystone taxa in the coupled network could promote N-P coupling, via stimulating growth and cross-feeding essential substrates in N and P cycling.

Moreover, we found that the ratio of positive to negative connections among taxa including putative keystone ones was lower in decoupled vs. coupled networks, suggesting that increasingly antagonistic interactions among taxa could be related to N-P decoupling. For example, there was a negative connection between *Sphingomonas* (a R-strategist) only correlated with N-cycling variables and an *unclassified Subgroup_17* genus (a potential keystone taxon in the decoupled network and a K-strategist) only correlated with P-cycling variables. Lu et al. (2024) also observed a negative relationship between the two genera, possibly because nutrient-poor conditions favor the K-strategist over the R-strategist. The resource competition-induced negative interactions among taxa involved in cycling of different nutrients might thus disrupt functional synergies in nutrient cycling, facilitating either N or P cycling, and ultimately leading to N-P decoupling. All in all, direct experimental evidence is needed to confirm cooperative and competitive interactions among taxa (especially potential keystone ones) and their mechanisms in facilitating N-P (de)coupling.

### 4.3 The functional divergence between microbial phyla and genera

Constructing networks at the phylum level simplifies analyses from thousands of taxa to a dozen groups and mitigates the challenge posed by rare taxa (Faust, 2021; Long et al., 2025), while constructing networks at the genus level aims at a finer taxonomic resolution (Stone et al., 2023). We believe the use of both phylum- and genus-level networks could provide a hierarchical and complementary perspective. Here, we found more cooperative interactions among coupled vs. decoupled taxa at both phylum and genus levels (Figure 6). The consistent interaction patterns across networks of different taxonomic levels have been observed in other ecosystems such as hot springs and wastewater (Zamkovaya et al., 2021; Hu et al., 2024). However, relationships between microbial taxa and nutrient cycling variables were often inconsistent between phylum and genus levels (Figures 4, 5). Only *Mortierellomycota* and the genera belonging to the phylum showed consistently positive relationships with nutrient cycling variables while *Mucoromycota* and the genera belonging to the phylum showed consistently negative relationships. The functional divergence among genera within the same phylum necessitates investigation at both broad and fine scales to capture the diverse mechanisms at play (Perez-Molphe-Montoya et al., 2022).

## 5 Conclusion

A total of 14 coupled phyla and 68 coupled genera were positively related to both N and P cycling, among which 5 coupled phyla and 24 coupled genera were generally enriched in sites 6–10 and 31–35. These taxa could facilitate coupled N and P cycling in these sites. Additionally, more cooperative interactions among coupled vs. decoupled taxa, as indicated by a higher ratio of positive to negative edges in the coupled vs. decoupled networks, highlights the importance of cooperative interactions among coupled taxa for coupled N-P cycling. Roles of interactions among putative keystone genera and network members in coupling and decoupling N and P cycling were partly confirmed by literature, although the involved metabolic pathways require further investigation. Thus, the coupled taxa might enhance N-P coupling both independently and through synergistic interactions. Overall, our study provides a good start to further examine potentials of these coupled taxa and their interactions in promoting both N and P cycling in forest ecosystems, which lays the groundwork for advancing forest ecosystem health in the context of environmental change. Although this study provides new insight into microbial contributions to N-P coupling, the findings remain constrained by system-specific conditions. Future work extending analysis across larger spatial and temporal scales will help validate and generalize these patterns.

## Data Availability

The datasets presented in this study can be found in online repositories. The names of the repository/repositories and accession number(s) can be found at: https://www.ncbi.nlm.nih.gov/ (PRJNA1330695, PRJNA1328234, and PRJNA1328344).

## References

[ref1] Ahash S. Manikandan K. Devi T. S. Elamathi S. Maragatham S. Subrahmaniyan K. (2025). Phosphate-solubilizing microorganisms for sustainable phosphorus management in rice. Rhizosphere 34:101096. doi: 10.1016/j.rhisph.2025.101096

[ref2] Akplo T. M. Kouelo Alladassi F. Zoundji M. C. C. Faye A. Hernández M. Yemadje P. L. . (2025). Phosphate solubilization and mobilization: bacteria–mycorrhiza interactions. Lett. Appl. Microbiol. 78:ovaf105. doi: 10.1093/lambio/ovaf105, 40736527

[ref3] An S. Li J. Du J. Feng L. Zhang L. Zhang X. . (2025). Coupled nitrogen and phosphorus cycles mediated by coordinated variations of functional microbes in industrial recirculating aquaculture system. Water Res. 280:123726. doi: 10.1016/j.watres.2025.123726, 40305950

[ref4] Banerjee S. Schlaeppi K. Van Der Heijden M. G. (2018). Keystone taxa as drivers of microbiome structure and functioning. Nat. Rev. Microbiol. 16, 567–576. doi: 10.1038/s41579-018-0024-1, 29789680

[ref5] Bennett E. M. Carpenter S. R. Caraco N. F. (2001). Human impact on erodable phosphorus and eutrophication: a global perspective: increasing accumulation of phosphorus in soil threatens rivers, lakes, and coastal oceans with eutrophication. Bio Science 51, 227–234. doi: 10.1641/0006-3568(2001)051[0227,HIOEPA]2.0.CO;2.

[ref6] Bergkemper F. Schöler A. Engel M. Lang F. Krüger J. Schloter M. . (2016). Phosphorus depletion in forest soils shapes bacterial communities towards phosphorus recycling systems. Environ. Microbiol. 18, 1988–2000. doi: 10.1111/1462-2920.13188, 26690731

[ref7] Bouyoucos G. J. (1962). Hydrometer method improved for making particle size analyses of soils^1^. Agron. J. 54, 464–465. doi: 10.2134/agronj1962.00021962005400050028x

[ref8] Callahan B. J. McMurdie P. J. Rosen M. J. Han A. W. Johnson A. J. A. Holmes S. P. (2016). DADA2: high-resolution sample inference from Illumina amplicon data. Nat. Methods 13, 581–583. doi: 10.1038/nmeth.3869, 27214047 PMC4927377

[ref9] Chadwick O. A. Derry L. A. Vitousek P. M. Huebert B. J. Hedin L. O. (1999). Changing sources of nutrients during four million years of ecosystem development. Nature 397, 491–497. doi: 10.1038/17276

[ref10] Chen W. Wang J. Chen X. Meng Z. Xu R. Duoji D. . (2022). Soil microbial network complexity predicts ecosystem function along elevation gradients on the Tibetan plateau. Soil Biol. Biochem. 172:108766. doi: 10.1016/j.soilbio.2022.108766

[ref11] Chen S. Yang D. Wei Y. He L. Li Z. Yang S. (2023). Changes in soil phosphorus availability and microbial community structures in rhizospheres of oilseed rapes induced by intercropping with white lupins. Microorganisms 11:326. doi: 10.3390/microorganisms11020326, 36838291 PMC9959241

[ref12] Cheng W. Wang Y. Wang Y. Hong L. Qiu M. Luo Y. . (2025). Aerospace mutagenized tea tree increases rhizospheric microorganisms, enhances nutrient conversion capacity and promotes growth. Plants 14:981. doi: 10.3390/plants14070981, 40219049 PMC11990241

[ref13] Cheng Y. Wang J. Wang J. Wang S. Chang S. X. Cai Z. . (2020). Nitrogen deposition differentially affects soil gross nitrogen transformations in organic and mineral horizons. Earth-Sci. Rev. 201:103033. doi: 10.1016/j.earscirev.2019.103033

[ref14] Cheng H. Yuan M. Tang L. Shen Y. Yu Q. Li S. (2022). Integrated microbiology and metabolomics analysis reveal responses of soil microorganisms and metabolic functions to phosphorus fertilizer on semiarid farm. Sci. Total Environ. 817:152878. doi: 10.1016/j.scitotenv.2021.152878 34998744

[ref15] Dai Z. Liu G. Chen H. Chen C. Wang J. Ai S. . (2020). Long-term nutrient inputs shift soil microbial functional profiles of phosphorus cycling in diverse agroecosystems. ISME J. 14, 757–770. doi: 10.1038/s41396-019-0567-9, 31827246 PMC7031380

[ref16] Delgado-Baquerizo M. Maestre F. T. Gallardo A. Bowker M. A. Wallenstein M. D. Quero J. L. . (2013). Decoupling of soil nutrient cycles as a function of aridity in global drylands. Nature 502, 672–676. doi: 10.1038/nature12670, 24172979

[ref17] Dong R. Yang S. Wang X. Xie L. Ma Y. Wang Y. . (2023). C: N: P stoichiometry in plant, soil and microbe in Sophora moorcroftiana shrubs across three sandy dune types in the middle reaches of the Yarlung Zangbo River. Front. Plant Sci. 13:1060686. doi: 10.3389/fpls.2022.1060686, 36714721 PMC9874299

[ref18] Eivazi F. Tabatabai M. A. (1988). Glucosidases and galactosidases in soils. Soil Biol. Biochem. 20, 601–606. doi: 10.1016/0038-0717(88)90141-1

[ref19] Fang X. Zheng R. Guo X. Fu Q. Zhang K. (2020). Responses of denitrification rate and denitrifying bacterial communities carrying *nirS* and *nirK* genes to grazing in peatland. J. Soil Sci. Plant Nutr. 20, 1249–1260. doi: 10.1007/s42729-020-00209-x

[ref20] Fang W. Zhu Y. Liang C. Shao S. Chen J. Qing H. . (2024). Deciphering differences in microbial community characteristics and main factors between healthy and root rot-infected *Carya cathayensis* rhizosphere soils. Front. Microbiol. 15:1448675. doi: 10.3389/fmicb.2024.1448675, 39588107 PMC11586369

[ref21] Faust K. (2021). Open challenges for microbial network construction and analysis. ISME J. 15, 3111–3118. doi: 10.1038/s41396-021-01027-4, 34108668 PMC8528840

[ref22] Feng X. Yan M. Gao G. He M. Zhang S. Wu M. (2025). Fertilization alters soil carbon metabolism by affecting the composition of modular microorganisms in paddy soil. Soil Use Manag. 41:e70046. doi: 10.1111/sum.70046

[ref23] Hayatsu M. Tago K. Saito M. (2008). Various players in the nitrogen cycle: diversity and functions of the microorganisms involved in nitrification and denitrification. Soil Sci. Plant Nutr. 54, 33–45. doi: 10.1111/j.1747-0765.2007.00195.x

[ref24] Hu H. Kristensen J. M. Herbold C. W. Pjevac P. Kitzinger K. Hausmann B. . (2024). Global abundance patterns, diversity, and ecology of *Patescibacteria* in wastewater treatment plants. Microbiome 12:55. doi: 10.1186/s40168-024-01769-1, 38493180 PMC10943839

[ref25] Huang Q. Long J. Zhang L. Qiu L. Xing S. (2025). Spatio-temporal variations of nitrogen to phosphorus ratios (N: P) in farmland topsoils of Fujian Province, China. J. Soil Sci. Plant Nutr. 25, 2897–2906. doi: 10.1007/s42729-025-02307-0

[ref26] Ji M. Williams T. J. Montgomery K. Wong H. L. Zaugg J. Berengut J. F. . (2021). *Candidatus* Eremiobacterota, a metabolically and phylogenetically diverse terrestrial phylum with acid-tolerant adaptations. ISME J. 15, 2692–2707. doi: 10.1038/s41396-021-00944-8, 33753881 PMC8397712

[ref27] Jian Z. Zeng L. Lei L. Frey B. Liu C. Shen Y. . (2025). Fungi stimulate organic phosphorus fraction transformation in subtropical masson pine plantation soils after nine years of thinning and understory removal. Ecol. Process. 14:23. doi: 10.1186/s13717-025-00586-0

[ref28] Jiao S. Peng Z. Qi J. Gao J. Wei G. (2021). Linking bacterial-fungal relationships to microbial diversity and soil nutrient cycling. mSystems 6:e01052-20. doi: 10.1128/mSystems.01052-20, 33758030 PMC8546990

[ref29] Jien S. Kuo Y. Liao C. Wu Y. Igalavithana A. D. Tsang D. C. W. . (2021). Effects of field scale in situ biochar incorporation on soil environment in a tropical highly weathered soil. Environ. Pollut. 272:116009. doi: 10.1016/j.envpol.2020.116009, 33257150

[ref30] Kang H. Gao H. Yu W. Yi Y. Wang Y. Ning M. (2018). Changes in soil microbial community structure and function after afforestation depend on species and age: case study in a subtropical alluvial island. Sci. Total Environ. 625, 1423–1432. doi: 10.1016/j.scitotenv.2017.12.180, 29996439

[ref31] Karimi B. Maron P. A. Chemidlin-Prevost Boure N. Bernard N. Gilbert D. Ranjard L. (2017). Microbial diversity and ecological networks as indicators of environmental quality. Environ. Chem. Lett. 15, 265–281. doi: 10.1007/s10311-017-0614-6

[ref32] Lacerda-Júnior G. V. Noronha M. F. Cabral L. Delforno T. P. de Sousa S. T. P. Fernandes-Júnior P. I. . (2019). Land use and seasonal effects on the soil microbiome of a Brazilian dry forest. Front. Microbiol. 10:648. doi: 10.3389/fmicb.2019.00648, 31024471 PMC6461016

[ref33] Lang M. Li J. Su W. Zou W. Liu Y. Chen X. (2022). Effects of long-term phosphorus application on *phoD* harboring bacterial community in calcareous soil. Acta Microbiol Sin. 62, 242–258. doi: 10.13343/j.cnki.wsxb.20210209

[ref34] Li H. Bi Q. Yang K. bo Lasson S. Zheng B. Cui L. . (2020). High starter phosphorus fertilization facilitates soil phosphorus turnover by promoting microbial functional interaction in an arable soil. J. Environ. Sci. 94, 179–185. doi: 10.1016/j.jes.2020.03.040, 32563482

[ref35] Li J. Lu J. Wang H. Fang Z. Wang X. Feng S. . (2021). A comprehensive synthesis unveils the mysteries of phosphate-solubilizing microbes. Biol. Rev. Camb. Philos. Soc. 96, 2771–2793. doi: 10.1111/brv.12779, 34288351 PMC9291587

[ref36] Li Y. Wang J. He L. Xu X. Wang J. Ren C. . (2022). Different mechanisms driving increasing abundance of microbial phosphorus cycling gene groups along an elevational gradient. iScience 25:105170. doi: 10.1016/j.isci.2022.105170, 36204265 PMC9529982

[ref37] Li P. Wu G. Li Y. Hu C. Ge L. Zheng X. . (2022). Long-term rice-crayfish-turtle co-culture maintains high crop yields by improving soil health and increasing soil microbial community stability. Geoderma 413:115745. doi: 10.1016/j.geoderma.2022.115745

[ref38] Liang X. S. Ma W. Hu J. X. Zhang B. C. Wang Z. W. Lv X. T. (2022). Extreme drought exacerbates plant nitrogen-phosphorus imbalance in nitrogen enriched grassland. Sci. Total Environ. 849:157916. doi: 10.1016/j.scitotenv.2022.157916 35963412

[ref39] Liu Z. Gu H. Yao Q. Jiao F. Hu X. Liu J. . (2024). Soil pH and carbon quality index regulate the biogeochemical cycle couplings of carbon, nitrogen and phosphorus in the profiles of Isohumosols. Sci. Total Environ. 922:171269. doi: 10.1016/j.scitotenv.2024.171269, 38423323

[ref40] Liu Y. Li X. Duan Y. Wang B. Wang W. Liu Z. . (2022). Effects of vegetation restoration on soil ecological stoichiometry in the eastern Kubuqi Desert. Arid Zone Res. 39, 924–932. doi: 10.13866/j.azr.2022.03.26

[ref41] Liu X. Pang L. Yue Y. Li H. Chatzisymeon E. Lu Y. . (2023). Insights into the shift of microbial community related to nitrogen cycle, especially N_2_O in vanadium-polluted soil. Environ. Pollut. 322:121253. doi: 10.1016/j.envpol.2023.121253, 36773688

[ref42] Liu X. Y. Wang H. Wang W. Cheng X. Wang Y. Li Q. . (2023). Nitrate determines the bacterial habitat specialization and impacts microbial functions in a subsurface karst cave. Front. Microbiol. 14:1115449. doi: 10.3389/fmicb.2023.1115449, 36846803 PMC9947541

[ref43] Liu F. Zeng J. Ding J. Wang C. He Z. Liu Z. . (2025). Microbially-driven phosphorus cycling and its coupling mechanisms with nitrogen cycling in mangrove sediments. Sci. Total Environ. 958:178118. doi: 10.1016/j.scitotenv.2024.178118, 39700989

[ref44] Long X. Li J. Liao X. Wang J. Zhang W. Wang K. . (2025). Stable soil biota network enhances soil multifunctionality in agroecosystems. Glob. Change Biol. 31:e70041. doi: 10.1111/gcb.70041, 39840664

[ref45] Lu Y. Gao Z. Zhu Y. Yao D. Wang X. (2024). Microbial community structure, diversity, and succession during decomposition of kiwifruit litters with different qualities. Microorganisms 12:2498. doi: 10.3390/microorganisms12122498, 39770701 PMC11727838

[ref46] Luo G. Ling N. Nannipieri P. Chen H. Raza W. Wang M. . (2017). Long-term fertilisation regimes affect the composition of the alkaline phosphomonoesterase encoding microbial community of a vertisol and its derivative soil fractions. Biol. Fertil. Soils 53, 375–388. doi: 10.1007/s00374-017-1183-3

[ref47] Lv H. Ding J. Zhang L. Wang C. Cai H. (2024). Zinc application facilitates the turnover of organic phosphorus in rice rhizosphere soil by modifying microbial communities. Plant Soil 498, 77–92. doi: 10.1007/s11104-022-05810-w

[ref48] Ma T. Yang K. Yang L. Zhu Y. Jiang B. Xiao Z. . (2024). Different rotation years change the structure and diversity of microorganisms in the nitrogen cycle, affecting crop yield. Appl. Soil Ecol. 193:105123. doi: 10.1016/j.apsoil.2023.105123

[ref49] Ma L. Zhang J. Li Z. Xin X. Guo Z. Wang D. . (2020). Long-term phosphorus deficiency decreased bacterial-fungal network complexity and efficiency across three soil types in China as revealed by network analysis. Appl. Soil Ecol. 148:103506. doi: 10.1016/j.apsoil.2020.103506

[ref50] Mahmoud R. S. Narisawa K. (2013). A new fungal endophyte, *Scolecobasidium humicola*, promotes tomato growth under organic nitrogen conditions. PLoS One 8:e78746. doi: 10.1371/journal.pone.0078746, 24223848 PMC3815298

[ref51] McIlroy S. J. Awata T. Nierychlo M. Albertsen M. Kindaichi T. Nielsen P. H. (2015). Characterization of the in *situ ecophysiology* of novel phylotypes in nutrient removal activated sludge treatment plants. PLoS One 10:e0136424. doi: 10.1371/journal.pone.0136424, 26340564 PMC4560404

[ref52] Mehlich A. (1953). Determination of P, ca, Mg, K, Na, and NH4. Raleigh, NC: North Carolina Soil Test Division, Mimeo.

[ref53] Mehrshad M. Salcher M. M. Okazaki Y. Nakano S. I. Šimek K. Andrei A. S. . (2018). Hidden in plain sight-highly abundant and diverse planktonic freshwater *Chloroflexi*. Microbiome 6:176. doi: 10.1186/s40168-018-0563-8, 30285851 PMC6169038

[ref54] Ministry of Environmental Protection of the People's Republic of China (2012). Soil-determination of ammonium, nitrite and nitrate by extraction with potassium chloride solution-spectrophotometric methods. Beijing: China Environmental Science Press.

[ref55] Miralles I. Ortega R. del Carmen Montero-Calasanz M. (2023). Functional and biotechnological potential of microbiome associated with soils colonised by cyanobacteria in drylands. Appl. Soil Ecol. 192:105076. doi: 10.1016/j.apsoil.2023.105076

[ref56] Mori H. Maruyama F. Kato H. Toyoda A. Dozono A. Ohtsubo Y. . (2014). Design and experimental application of a novel non-degenerate universal primer set that amplifies prokaryotic 16S rRNA genes with a low possibility to amplify eukaryotic rRNA genes. DNA Res. 21, 217–227. doi: 10.1093/dnares/dst052, 24277737 PMC3989492

[ref57] Mosley O. E. Gios E. Handley K. M. (2024). Implications for nitrogen and Sulphur cycles: phylogeny and niche-range of *Nitrospirota* in terrestrial aquifers. ISME Commun. 4:ycae047. doi: 10.1093/ismeco/ycae047, 38650708 PMC11033732

[ref58] National Soil Survey Office (1990). The Chinese soil census technology. Beijing: China Agricultural Press.

[ref59] Nie Y. Wang M. Zhang W. Ni Z. Hashidoko Y. Shen W. (2018). Ammonium nitrogen content is a dominant predictor of bacterial community composition in an acidic forest soil with exogenous nitrogen enrichment. Sci. Total Environ. 624, 407–415. doi: 10.1016/j.scitotenv.2017.12.142, 29262382

[ref60] Pang F. Li Q. Solanki M. K. Wang Z. Xing Y. Dong D. (2024). Soil phosphorus transformation and plant uptake driven by phosphate-solubilizing microorganisms. Front. Microbiol. 15:1383813. doi: 10.3389/fmicb.2024.1383813, 38601943 PMC11005474

[ref61] Park S. You Y. Kim Y. Kwon E. Ansari A. Kim S. M. . (2022). *Ureaplasma* and *Prevotella* colonization with *Lactobacillus* abundance during pregnancy facilitates term birth. Sci. Rep. 12:10148. doi: 10.1038/s41598-022-13871-1, 35710793 PMC9203766

[ref62] Peñuelas J. Janssens I. A. Ciais P. Obersteiner M. Sardans J. (2020). Anthropogenic global shifts in biospheric N and P concentrations and ratios and their impacts on biodiversity, ecosystem productivity, food security, and human health. Glob. Chang. Biol. 26, 1962–1985. doi: 10.1111/gcb.14981, 31912629

[ref63] Peñuelas J. Poulter B. Sardans J. Ciais P. der Van Velde M. Bopp L. . (2013). Human-induced nitrogen–phosphorus imbalances alter natural and managed ecosystems across the globe. Nat. Commun. 4:2934. doi: 10.1038/ncomms3934, 24343268

[ref64] Peñuelas J. Sardans J. (2022). The global nitrogen-phosphorus imbalance. Science 375, 266–267. doi: 10.1126/science.abl4827, 35050668

[ref65] Perez-Molphe-Montoya E. Küsel K. Overholt W. A. (2022). Redefining the phylogenetic and metabolic diversity of phylum *Omnitrophota*. Environ. Microbiol. 24, 5437–5449. doi: 10.1111/1462-2920.16170, 36123312

[ref66] Pessi I. S. Delmont T. O. Zehr J. P. Hultman J. (2024). Discovery of Eremiobacterota with *nifH* homologues in tundra soil. Environ. Microbiol. Rep. 16:e13277. doi: 10.1111/1758-2229.13277, 38881156 PMC11180709

[ref67] R Core Team (2024). R: A language and environment for statistical computing. Vienna, Austria: R Foundation for Statistical Computing.

[ref68] Ren P. Sun A. Jiao X. Chen Q. L. Hu H. W. (2025). The relationship between protist consumers and soil functional genes under long-term fertilization. Sci. Total Environ. 966:178658. doi: 10.1016/j.scitotenv.2025.178658, 39904217

[ref69] Sang Y. Jin L. Zhu R. Yu X.-Y. Hu S. Wang B. . (2022). Phosphorus-solubilizing capacity of *Mortierella* species isolated from rhizosphere soil of a poplar plantation. Microorganisms 10:2361. doi: 10.3390/microorganisms10122361, 36557615 PMC9785298

[ref70] Sharma S. B. Sayyed R. Z. Trivedi M. H. Gobi T. A. (2013). Phosphate solubilizing microbes: sustainable approach for managing phosphorus deficiency in agricultural soils. Springerplus 2:587. doi: 10.1186/2193-1801-2-587, 25674415 PMC4320215

[ref71] Shen Y. Tian D. Jiang L. Wang J. Chen X. Li Y. . (2019). Different responses and links of N: P ratio among ecosystem components under nutrient addition in a temperate forest. J. Geophys. Res. Biogeosci. 124, 3158–3167. doi: 10.1029/2019JG005080

[ref72] Sinsabaugh R. L. Lauber C. L. Weintraub M. N. Ahmed B. Allison S. D. Crenshaw C. . (2008). Stoichiometry of soil enzyme activity at global scale. Ecol. Lett. 11, 1252–1264. doi: 10.1111/j.1461-0248.2008.01245.x, 18823393

[ref73] Stone B. W. Dijkstra P. Finley B. K. Fitzpatrick R. Foley M. M. Hayer M. . (2023). Life history strategies among soil bacteria—dichotomy for few, continuum for many. ISME J. 17, 611–619. doi: 10.1038/s41396-022-01354-0, 36732614 PMC10030646

[ref74] Stosiek N. Terebieniec A. Ząbek A. Młynarz P. Cieśliński H. Klimek-Ochab M. (2019). *N*-phosphonomethylglycine utilization by the psychrotolerant yeast *Solicoccozyma terricola* M 3.1.4. Bioorg. Chem. 93:102866. doi: 10.1016/j.bioorg.2019.03.040, 30902434

[ref75] Sun Q. Chen J. Liao X. Huang X. Liu J. (2024). Identification of keystone taxa in rhizosphere microbial communities using different methods and their effects on compounds of the host *Cinnamomum migao*. Sci. Total Environ. 926:171952. doi: 10.1016/j.scitotenv.2024.171952, 38537823

[ref76] Tao D. Gao Y. (2023). Advances on the strategies of soil phosphate solubilizing microorganisms to promote plant phosphorus uptake. Acta Ecol. Sin. 43, 4390–4399. doi: 10.5846/stxb202111193253

[ref77] Tian H. Chen G. Zhang C. Melillo J. M. Hall C. A. S. (2010). Pattern and variation of C:N:P ratios in China’s soils: a synthesis of observational data. Biogeochemistry 98, 139–151. doi: 10.1007/s10533-009-9382-0

[ref78] Truu M. Nõlvak H. Ostonen I. Oopkaup K. Maddison M. Ligi T. . (2020). Soil bacterial and archaeal communities and their potential to perform N-cycling processes in soils of boreal forests growing on well-drained peat. Front. Microbiol. 11:591358. doi: 10.3389/fmicb.2020.591358, 33343531 PMC7744593

[ref79] Wang H. Chen Y. Du L. Liu H. Wen F. (2023). Effects of modifier application and *Koelreuteria Paniculata* planting on the microbial community of a manganese slag. Chin. J. Environ. Eng. 17, 2544–2555. doi: 10.12030/j.cjee.202303056

[ref80] Wang X. Guo H. Wang J. He P. Kuzyakov Y. Ma M. . (2024). Microbial phosphorus-cycling genes in soil under global change. Glob. Change Biol. 30:e17281. doi: 10.1111/gcb.17281, 38619550

[ref81] Wang L. Hong Y. Li H. Li J. (2022). Bacterial community structure and diversity in the rhizosphere soil of *Elymus sibiricus* Linn., *Elymus canadensis* L. and the first hybrid generation. Chin. J. North Agric. 50, 24–33. doi: 10.12190/j.issn.2096-1197.2022.05.04

[ref82] Wang F. Luo Y. Huang W. Chen J. Cao J. Feng L. . (2023). Unveiling polyhexamethylene guanidine regulating behaviors on methane generation in a sludge anaerobic digester: interactive process, microbial metabolic trait interference, and adaptive mechanisms. ACS ES&T Eng. 4, 717–727. doi: 10.1021/acsestengg.3c00458

[ref83] Wang Z. Ruan X. Li R. Zhang Y. (2024). Microbial interaction patterns and nitrogen cycling regularities in lake sediments under different trophic conditions. Sci. Total Environ. 907:167926. doi: 10.1016/j.scitotenv.2023.167926, 37863216

[ref84] Wang W. Wang C. Sardans J. Min Q. Zeng C. Tong C. . (2015). Agricultural land use decouples soil nutrient cycles in a subtropical riparian wetland in China. Catena 133, 171–178. doi: 10.1016/j.catena.2015.05.003

[ref85] Wang X. Yao B. Yang H. Mou X. Li Y. Li Y. (2025). Microbial drivers of soil C: N: P stoichiometry dynamics during ecological restoration in sandy ecosystems. Appl. Soil Ecol. 213:106321. doi: 10.1016/j.apsoil.2025.106321

[ref86] Wu Q. Chen D. Zhou W. Zhang X. Ao J. (2022). Long-term fertilization has different impacts on bacterial communities and phosphorus forms in sugarcane rhizosphere and bulk soils under low-P stress. Front. Plant Sci. 13:1019042. doi: 10.3389/fpls.2022.1019042, 36212295 PMC9539793

[ref87] Xu X. Thornton P. E. Post W. M. (2013). A global analysis of soil microbial biomass carbon, nitrogen and phosphorus in terrestrial ecosystems. Glob. Ecol. Biogeogr. 22, 737–749. doi: 10.1111/geb.12029

[ref88] Yu Q. Ni X. Hagedorn F. Penuelas J. Sardans J. Li M. H. . (2025). Field experiments and a meta-analysis reveal a minor influence of nitrogen addition on phosphorus fractions in forests. Glob. Change Biol. 31:e70156. doi: 10.1111/gcb.70156, 40237226

[ref89] Yu M. Tao Y. Liu W. Xing W. Liu G. Wang L. . (2020). C, N, and P stoichiometry and their interaction with different plant communities and soils in subtropical riparian wetlands. Environ. Sci. Pollut. Res. 27, 1024–1034. doi: 10.1007/s11356-019-07004-x, 31820250

[ref90] Yu H. Wang F. Shao M. Huang L. Xie Y. Xu Y. . (2021). Effects of rotations with legume on soil functional microbial communities involved in phosphorus transformation. Front. Microbiol. 12:661100. doi: 10.3389/fmicb.2021.661100, 34659135 PMC8519609

[ref91] Yuan Z. Chen H. Y. (2015). Decoupling of nitrogen and phosphorus in terrestrial plants associated with global changes. Nat. Clim. Chang. 5, 465–469. doi: 10.1038/nclimate2549

[ref92] Yun Y. Wang H. Man B. Xiang X. Zhou J. Qiu X. . (2016). The relationship between pH and bacterial communities in a single karst ecosystem and its implication for soil acidification. Front. Microbiol. 7:1955. doi: 10.3389/fmicb.2016.01955, 28018299 PMC5159436

[ref93] Zamkovaya T. Foster J. S. de Crécy-Lagard V. Conesa A. (2021). A network approach to elucidate and prioritize microbial dark matter in microbial communities. ISME J. 15, 228–244. doi: 10.1038/s41396-020-00777-x, 32963345 PMC7852563

[ref94] Zhang Y. Cai Z. Zhang J. Müller C. (2020). C: N ratio is not a reliable predictor of N_2_O production in acidic soils after a 30-day artificial manipulation. Sci. Total Environ. 725:138427. doi: 10.1016/j.scitotenv.2020.138427, 32464751

[ref95] Zhang N. Guo R. Song P. Guo J. Gao Y. (2013). Effects of warming and nitrogen deposition on the coupling mechanism between soil nitrogen and phosphorus in Songnen meadow steppe, northeastern China. Soil Biol. Biochem. 65, 96–104. doi: 10.1016/j.soilbio.2013.05.015

[ref96] Zhang H. Jiang N. Zhang S. Zhu X. Wang H. Xiu W. . (2024). Soil bacterial community composition is altered more by soil nutrient availability than pH following long-term nutrient addition in a temperate steppe. Front. Microbiol. 15:1455891. doi: 10.3389/fmicb.2024.1455891, 39345260 PMC11427344

[ref97] Zhang C. Lei S. Wu H. Liao L. Wang X. Zhang L. . (2024). Simplified microbial network reduced microbial structure stability and soil functionality in alpine grassland along a natural aridity gradient. Soil Biol. Biochem. 191:109366. doi: 10.1016/j.soilbio.2024.109366

[ref98] Zhang Z. Wang B. Buyantuev A. He X. Gao W. Wang Y. . (2019). Urban agglomeration of Kunming and Yuxi cities in Yunnan, China: the relative importance of government policy drivers and environmental constraints. Landsc. Ecol. 34, 663–679. doi: 10.1007/s10980-019-00790-2

[ref99] Zhang X. Zhan Y. Zhang H. Wang R. Tao X. Zhang L. . (2021). Inoculation of phosphate-solubilizing bacteria (*Bacillus*) regulates microbial interaction to improve phosphorus fractions mobilization during kitchen waste composting. Bioresour. Technol. 340:125714. doi: 10.1016/j.biortech.2021.125714, 34371333

[ref100] Zhou L. Jiang J. Xie J. Chen Y. Guo H. Dai W. . (2025). Long-term nitrogen addition changes phosphorus availability and reshapes phosphate-solubilizing bacterial community in purple soil in Southwest China. Environ. Technol. Innov. 40:104353. doi: 10.1016/j.eti.2025.104353

[ref101] Zhu Z. Ding J. Du R. Zhang Z. Guo J. Li X. . (2024). Systematic tracking of nitrogen sources in complex river catchments: machine learning approach based on microbial metagenomics. Water Res. 253:121255. doi: 10.1016/j.watres.2024.121255, 38341971

[ref102] Zhu J. Li M. Whelan M. (2018). Phosphorus activators contribute to legacy phosphorus availability in agricultural soils: a review. Sci. Total Environ. 612, 522–537. doi: 10.1016/j.scitotenv.2017.08.095, 28865270

[ref103] Zhu J. Niu W. Zhang Z. Siddique K. H. Sun D. Yang R. (2022). Distinct roles for soil bacterial and fungal communities associated with the availability of carbon and phosphorus under aerated drip irrigation. Agric. Water Manag. 274:107925. doi: 10.1016/j.agwat.2022.107925

