# Supplementary material for: Coupled N and P cycling as driven by microbial taxa and interactions
Source: Front Microbiol. 2026 Jan 8;16:1743883. doi: 10.3389/fmicb.2025.1743883 (PMC12823974; doi:10.3389/fmicb.2025.1743883)
Supplement: Supplementary file 1 [file Table_1.docx]

Supplementary Information for

**Coupled N and P cycling as driven by microbial taxa and interaction**

**Supplementary Table 1. Environmental and soil physicochemical properties and soil organic matter chemical composition at 35 forest sites in Yunnan**

|  | Latitude (oN) | Longitude (oE) | Altitude (m) | MAP (mm) | MAT (°C) | Moisture (%) | pH (in water) | Sand (%) | Silt  (%) | Clay  (%) | SOC (%) | TN  (%) | TP  (%) |
| --- | --- | --- | --- | --- | --- | --- | --- | --- | --- | --- | --- | --- | --- |
| 1 | 25.03 | 102.90 | 2137 | 935 | 14.05 | 24.78 | 5.57 | 14.00 | 37.00 | 50.60 | 3.23 | 0.18 | 0.11 |
| 2 | 25.03 | 102.90 | 2159 | 935 | 14.05 | 28.12 | 6.46 | 26.30 | 40.60 | 33.40 | 3.59 | 0.21 | 0.23 |
| 3 | 25.02 | 102.90 | 2156 | 935 | 14.05 | 23.95 | 6.68 | 18.40 | 49.70 | 33.30 | 3.56 | 0.19 | 0.17 |
| 4 | 25.02 | 102.90 | 2131 | 935 | 14.05 | 28.02 | 7.82 | 22.80 | 41.70 | 35.50 | 2.62 | 0.16 | 0.15 |
| 5 | 25.01 | 102.90 | 2152 | 935 | 14.05 | 20.81 | 7.87 | 33.60 | 44.00 | 20.70 | 4.77 | 0.26 | 0.12 |
| 6 | 24.77 | 102.93 | 2671 | 991 | 11.93 | 58.62 | 5.26 | 53.20 | 30.50 | 13.10 | 13.73 | 0.93 | 0.19 |
| 7 | 24.77 | 102.93 | 2672 | 991 | 11.93 | 71.92 | 5.30 | 33.20 | 29.80 | 13.20 | 15.62 | 1.03 | 0.24 |
| 8 | 24.77 | 102.93 | 2656 | 991 | 11.93 | 64.78 | 5.35 | 50.20 | 29.40 | 13.20 | 14.37 | 1.00 | 0.22 |
| 9 | 24.76 | 102.92 | 2666 | 991 | 11.93 | 68.64 | 5.42 | 52.10 | 33.00 | 11.10 | 13.05 | 0.88 | 0.18 |
| 10 | 24.76 | 102.92 | 2634 | 991 | 11.93 | 69.14 | 5.38 | 62.40 | 24.30 | 8.10 | 14.70 | 1.00 | 0.17 |
| 11 | 24.71 | 102.93 | 2010 | 959 | 13.96 | 7.01 | 5.54 | 60.40 | 21.20 | 22.30 | 1.42 | 0.07 | 0.02 |
| 12 | 24.71 | 102.93 | 2030 | 959 | 13.96 | 8.27 | 5.46 | 58.80 | 21.80 | 20.30 | 1.35 | 0.07 | 0.01 |
| 13 | 24.72 | 102.94 | 2040 | 959 | 13.96 | 8.29 | 5.99 | 49.40 | 32.10 | 21.20 | 1.59 | 0.09 | 0.02 |
| 14 | 24.73 | 102.94 | 2036 | 959 | 13.96 | 11.87 | 6.35 | 50.40 | 28.20 | 23.30 | 1.73 | 0.10 | 0.02 |
| 15 | 24.74 | 102.94 | 2099 | 959 | 13.96 | 12.09 | 5.97 | 43.00 | 31.00 | 26.70 | 2.17 | 0.13 | 0.02 |
| 16 | 24.62 | 102.82 | 2142 | 931 | 14.69 | 12.69 | 6.05 | 38.80 | 26.00 | 35.50 | 1.43 | 0.10 | 0.04 |
| 17 | 24.63 | 102.82 | 2158 | 968 | 13.76 | 15.41 | 5.97 | 33.80 | 34.20 | 30.70 | 2.93 | 0.19 | 0.05 |
| 18 | 24.63 | 102.81 | 2213 | 968 | 13.76 | 9.24 | 5.53 | 29.00 | 51.30 | 20.10 | 1.89 | 0.13 | 0.05 |
| 19 | 24.63 | 102.81 | 2238 | 968 | 13.76 | 13.90 | 5.41 | 23.80 | 47.40 | 29.20 | 1.85 | 0.13 | 0.05 |
| 20 | 24.63 | 102.81 | 2241 | 968 | 13.76 | 14.54 | 5.67 | 29.40 | 38.20 | 33.00 | 1.41 | 0.13 | 0.06 |
| 21 | 24.56 | 102.73 | 2305 | 974 | 13.42 | 25.59 | 6.44 | 22.50 | 36.10 | 42.00 | 3.58 | 0.25 | 0.17 |
| 22 | 24.55 | 102.74 | 2237 | 974 | 13.42 | 24.26 | 6.7 | 23.30 | 39.70 | 37.30 | 2.53 | 0.20 | 0.20 |
| 23 | 24.51 | 102.73 | 2224 | 974 | 13.42 | 29.54 | 5.89 | 20.50 | 34.80 | 45.10 | 3.52 | 0.23 | 0.14 |
| 24 | 24.54 | 102.75 | 2236 | 971 | 13.57 | 20.68 | 6.59 | 32.30 | 25.00 | 44.70 | 3.77 | 0.24 | 0.11 |
| 25 | 24.54 | 102.76 | 2199 | 953 | 13.92 | 22.33 | 6.82 | 24.80 | 38.60 | 36.30 | 5.21 | 0.35 | 0.12 |
| 26 | 24.45 | 103.00 | 1829 | 958 | 14.66 | 5.55 | 5.88 | 43.60 | 34.40 | 22.30 | 1.47 | 0.09 | 0.02 |
| 27 | 24.46 | 103.01 | 1883 | 958 | 14.66 | 8.17 | 6.87 | 46.90 | 36.50 | 19.30 | 1.50 | 0.09 | 0.03 |
| 28 | 24.45 | 103.03 | 1908 | 958 | 14.66 | 14.13 | 7.48 | 42.60 | 35.20 | 22.20 | 2.01 | 0.11 | 0.04 |
| 29 | 24.49 | 103.05 | 1994 | 917 | 15.62 | 17.05 | 5.79 | 38.10 | 37.50 | 23.50 | 2.08 | 0.12 | 0.02 |
| 30 | 24.51 | 103.06 | 1919 | 915 | 15.90 | 30.99 | 5.35 | 58.10 | 27.30 | 17.60 | 2.31 | 0.14 | 0.02 |
| 31 | 24.68 | 103.15 | 1970 | 927 | 14.87 | 30.54 | 5.27 | 20.80 | 46.00 | 34.60 | 3.75 | 0.23 | 0.05 |
| 32 | 24.68 | 103.15 | 1993 | 927 | 14.87 | 34.38 | 4.73 | 26.40 | 42.30 | 32.40 | 3.65 | 0.24 | 0.05 |
| 33 | 24.69 | 103.15 | 1992 | 927 | 14.87 | 36.25 | 5.37 | 46.70 | 33.70 | 21.10 | 4.81 | 0.30 | 0.06 |
| 34 | 24.69 | 103.15 | 2002 | 927 | 14.87 | 16.10 | 5.21 | 53.80 | 22.50 | 24.00 | 3.49 | 0.18 | 0.04 |
| 35 | 24.70 | 103.15 | 2003 | 927 | 14.87 | 21.06 | 5.32 | 27.90 | 45.20 | 27.00 | 4.18 | 0.30 | 0.09 |

MAP, mean annual precipitation; MAT, mean annual temperature. The climatic data is an average from 1970 to 2020 downloaded by worldclim (https://worldclim.org/).

**Supplementary Table 2. Number of nodes and links in N-P coupled and N-P decoupled networks**

|  | group | Phylum level | | Genus level | |
| --- | --- | --- | --- | --- | --- |
|  |  | N-P coupled | N-P decoupled | N-P coupled | N-P decoupled |
|  | B node | 7 | 6 | 34 | 29 |
|  | F node | 7 | 1 | 25 | 9 |
|  | P node | 0 | 4 | 5 | 6 |
| B-B link | Positive | 2 | 1 | 67 | 65 |
|  | Negative | 2 | 0 | 14 | 34 |
|  | Total | 4 | 1 | 81 | 99 |
| B-F link | Positive | 3 | 0 | 43 | 7 |
|  | Negative | 0 | 0 | **1** | **5** |
|  | Total | 3 | 0 | 44 | 12 |
| B-P link | Positive | 0 | 3 | 14 | 21 |
|  | Negative | **0** | **2** | **9** | **8** |
|  | Total | 0 | 5 | 23 | 29 |
| F-F link | Positive | 0 | 0 | 21 | 0 |
|  | Negative | 1 | 0 | 0 | 0 |
|  | Total | 1 | 0 | 21 | 0 |
| F-P link | Positive | 0 | 0 | 4 | 1 |
|  | Negative | **0** | **0** | **0** | **1** |
|  | Total | 0 | 0 | 4 | 2 |
| P-P link | Positive | 0 | 0 | 1 | 2 |
|  | Negative | 0 | 1 | 0 | 2 |
|  | Total | 0 | 1 | 1 | 4 |

B, bacteria; F, fungi; P, *phoD-*harboring bacteria

| 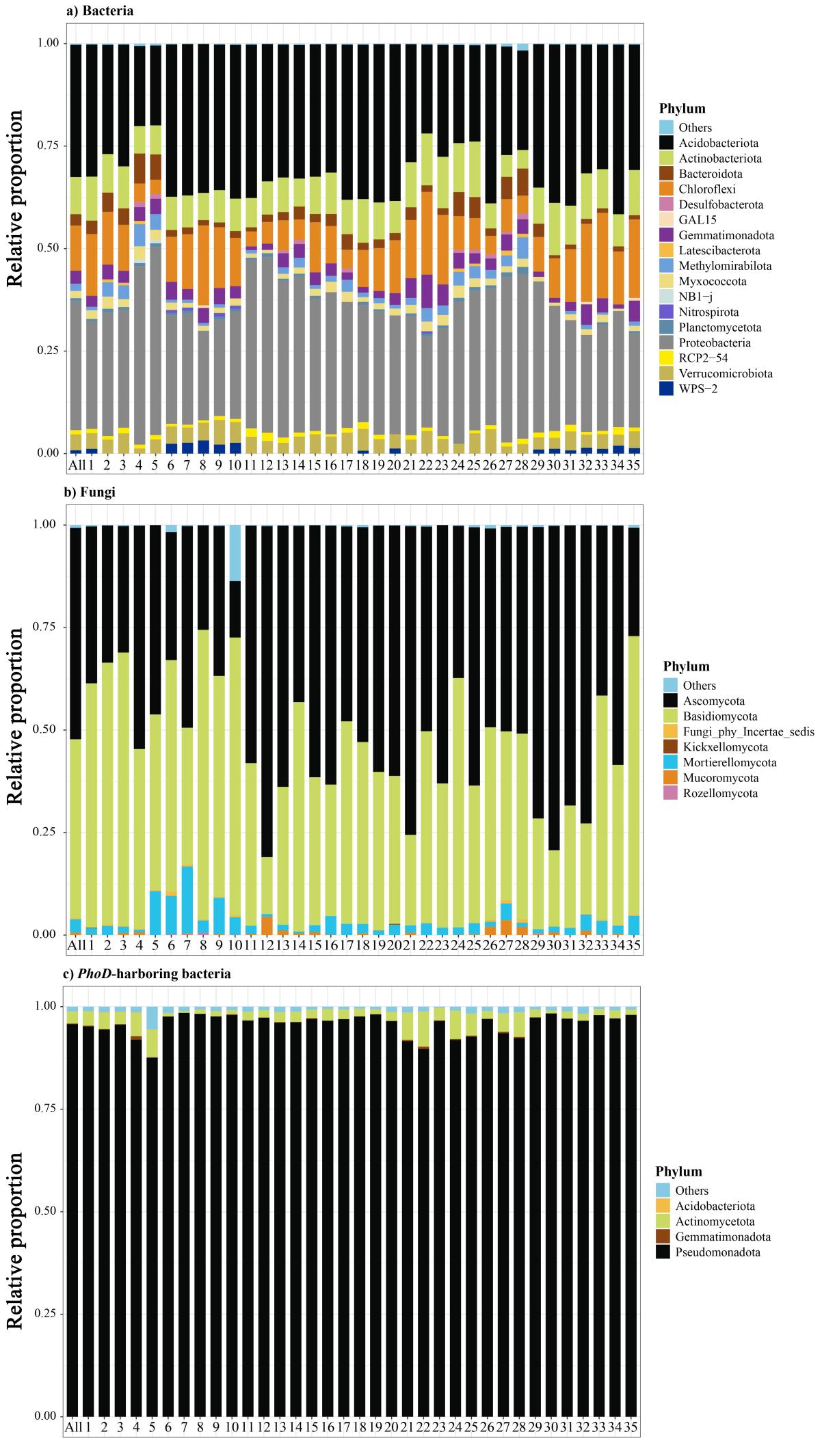 |
| --- |
| **Supplementary Figure 1.** Relative abundances of dominant a) bacterial, b) fungal, and c) *phoD*-harboring phyla (relative abundance > 0.1%) across 35 sites. “All” indicates site-averaged abundances. |
